# Understanding Remission of Long-Term Conditions Through Electronic Health Records: Scoping Review

**DOI:** 10.2196/80796

**Published:** 2026-05-19

**Authors:** Hilda Hounkpatin, Benjamin Barton, Margaret Ogden, Rohini Mathur, Beth Stuart, Hajira Dambha-Miller

**Affiliations:** 1 Primary Care Research Centre, University of Southampton, Southampton, SO16 5ST, United Kingdom, 44 2380590047; 2 Wolfson Institute of Population Health, Queen Mary University of London, London, United Kingdom

**Keywords:** remission, resolution, long-term conditions, electronic health records, scoping review

## Abstract

**Background:**

Multiple long-term conditions (MLTCs) require complex and prolonged treatment regimens. Remission in long-term conditions (LTCs) is important for understanding disease progression and evaluating treatment effectiveness. Electronic health records (EHRs) are increasingly used to monitor clinical outcomes, but how remission is defined within EHRs remains unclear.

**Objective:**

This study aimed to summarize and collate the previous literature on how remission of LTCs has been defined in EHRs.

**Methods:**

Systematic electronic searches were performed on OVID MEDLINE, Embase, CINAHL EBSCO, the Cochrane Library, and the Bielefeld Academic Search Engine for eligible studies published from inception to November 27, 2025. Quantitative studies, published in any language, on adult populations, and using EHRs to assess remission of LTCs, were eligible for inclusion. Studies that did not clearly define remission and studies on cancer remission were excluded. Data were extracted from each eligible study using a structured table. Risk of bias was not assessed, in line with scoping review methodology. A narrative approach was taken to summarize and present data from the included studies. The number and characteristics of studies were described, both overall and by condition. Findings were discussed with clinicians and data experts to ensure applicability in clinical practice.

**Results:**

Ninety-one studies were included. Sample sizes ranged from 12 to 72.9 million adults. Studies were conducted in 18 countries, with the majority being from the United States. The majority of included studies used a cohort study design. Studies assessed how remission was defined in 12 LTCs, including inflammatory bowel disease (41/91, 45.1%), type 2 diabetes (n=15, 16.5%), depression (n=15, 16.5%), alcohol or drug misuse (n=8, 8.8%), asthma (n=3, 3.3%), multiple sclerosis (n=3, 3.3%), epilepsy (n=1, 1.1%), anemia (n=1, 1.1%), chronic kidney disease (n=1, 1.1%), autoimmune pancreatitis (n=1, 1.1%), hypertension (n=1, 1.1%), heart failure (n=1, 1.1%), and MLTC (n=1, 1.1%). Remission was typically defined using a combination of clinical codes (n=7, 7.7%), validated rating scales (n=56, 61.5%), biochemical markers (n=29, 31.9%), absence of symptoms (n=10, 11%), absence of condition-specific events (eg, hospital admissions; n=4, 4.4%), and cessation of pharmacological treatments (n=26, 28.6%). There was substantial variation in the criteria and duration of follow-up used to define remission across studies.

**Conclusions:**

This review demonstrates that remission of LTCs can be identified and operationalized within EHRs, although remission criteria varied across studies. The review extends the literature on remission in EHRs by combining evidence synthesis and consultation with clinical and data experts to propose standardized comprehensive definitions to reliably define and implement remission of multiple LTCs in EHR-based research. This will allow cross-study comparisons and present an opportunity to advance understanding of disease trajectories and improve evaluation and monitoring of patient outcomes. Further research may apply, compare, and evaluate standardized definitions across different data sources to assess generalizability and further improve our understanding of remission of LTCs.

## Introduction

Multiple long-term conditions (MLTCs)—the presence of 2 or more long-term conditions within an individual [1]—are a major challenge to public health, as their management often involves prolonged treatment and care [2]. People living with MLTCs frequently face high treatment burden, reduced quality of life, and functional decline and are at increased risk of mortality and extensive health and social care use [3-8]. MLTCs are associated with worse health outcomes, with risks of mortality and hospitalization escalating sharply for individuals with 4 or more long-term conditions (LTCs) [9,10]. Effective management of MLTCs remains an unmet research priority, and there is a pressing need to identify new strategies to improve care and outcomes for individuals living with these conditions [11].

For many LTCs, remission—defined as the resolution or cessation of the disease—marks a positive clinical outcome, whether temporary or permanent. Evidence suggests that remission in various LTCs is associated with improved health outcomes [12-16]. For example, previous research using a population-based cohort in South England found that 19.1% of people living with type 2 diabetes achieved remission within a 2-year follow-up period, and those who achieved remission had a lower risk of cardiovascular outcomes and mortality [13]. Similarly, a national Swedish study found that 39.3% of people living with hypertension achieved remission during a 2-year follow-up period, and people who achieved remission had half the risk of developing cardiovascular events and mortality compared to those who did not [14]. Studies have also reported favorable clinical outcomes for people who experience remission in other conditions, such as depression, cancer, or heart disease [15,16]. These previous studies suggest that efforts to support people living with MLTCs to achieve remission could improve their health. However, this research has largely focused on specific conditions (and populations with individual conditions), making it less clear how remission of any LTC in the context of MLTCs may relate to outcomes.

Furthermore, some of these previous studies have identified remission using survey or interview studies [15,16], and it is therefore unclear how remission can be identified in electronic health records (EHRs). The study of disease and MLTCs has increasingly relied on EHRs, which have become essential tools for tracking patient health over time. EHRs provide valuable insights into disease progression and are central to the ongoing efforts to improve clinical decision-making, monitoring, and health policy [17-19]. However, there is limited research on remission in the context of MLTCs. This may be due to a lack of a consistent definition or method for defining and tracking remission of LTCs within EHRs, making the study of remission in EHRs challenging. Variation in clinical coding practices, the lack of dedicated remission fields, and differing interpretations of disease remission all further hinder comparability across studies. A consensus on the definition of remission of LTCs in EHRs is crucial to advancing MLTC research (eg, through allowing accurate estimates of MLTC prevalence and associations) and improving care (eg, tracking remission outcomes accurately), both in the United Kingdom and nationally.

As the use of EHRs in clinical research grows, it is timely to examine how remission of LTCs is defined and tracked within these systems. We conducted a scoping review, as this methodology allows for mapping the existing literature on the definition and identification of remission in EHRs and highlighting gaps in the research. For this review, we used the term remission to refer to either remission or resolution. The decision to use the term “remission” was guided by our public advisory group (people with lived experience of MLTC) who felt that many of their conditions could be controlled but not permanently resolved. Our public advisory group consisted of people from diverse backgrounds (in terms of sex, ethnicity, and socioeconomic position) and health conditions and have provided input at each stage of our study design. This scoping review aimed to collate and summarize the existing literature on how remission of LTCs that are amenable to remission has been defined and evaluated in studies using EHRs.

## Methods

### Review Approach

The study followed a scoping review methodology outlined by the PRISMA-ScR (Preferred Reporting Items for Systematic Reviews and Meta-Analyses extension for Scoping Reviews) guidelines [20,21]. For the purpose of this review, MLTCs were defined based on a consensus process involving experts in the field and reported in detail previously [22,23]. This definition includes 56 LTCs selected for their chronic, long-term impact on health, requiring ongoing management or treatment, based on established diagnostic criteria, significant population prevalence, and known effects on morbidity and mortality. The list of conditions is provided in Table S1 in Multimedia Appendix 1.

### Protocol and Registration

A review protocol for this study does not exist in any public domain.

### Information Sources

Systematic searches were conducted in 5 electronic databases: OVID MEDLINE, Embase, CINAHL, the Cochrane Database of Systematic Reviews, and Bielefeld Academic Search Engine on November 27, 2025, for studies published from inception to date. These databases were selected to capture a range of relevant studies in the biomedical, nursing, and behavioral sciences literature.

### Search Strategy

The search strategy is reported in line with the PRISMA-S guidelines [24]. Terms to include in our search strategy were identified based on previous similar or relevant studies, searches for MeSH (Medical Subject Headings) terms and keywords or index terms in each database, and support from a librarian at the University of Southampton. Initial searches were conducted in Ovid MEDLINE and Embase. Both databases were searched for MeSH terms and free-text keywords related to “remission,” “resolution,” and “electronic health records” or “electronic medical records,” and “chronic disease” or “long-term condition.” Searches were adjusted for both databases according to their specific indexing. We screened the first 100 records captured in each database to identify key terms used in the title and abstracts of these records. We then refined our search strategy based on our findings, including any additional terms identified in our initial screening. We applied our refined search strategy to each of our 5 databases, adapting the search strategy as required. The search strategies for each database are available in Multimedia Appendix 2. Reference lists of included studies were not searched for additional papers, and study authors were not contacted for any additional information. Gray literature searches were conducted mainly through Bielefeld Academic Search Engine database and Google Scholar searches to identify any additional relevant references (eg, conference abstracts and theses). We did not contact authors to identify additional sources.

### Eligibility Criteria

Quantitative studies were eligible for inclusion if they were conducted using EHRs, focused on an adult (aged ≥18 y) population, and assessed 1 or more of the 56 LTCs agreed by consensus work within the definition of MLTC [22,23]. In line with most existing research on MLTC, an adult population was selected as MLTC is more common in adults [25], and the criteria used to define remission may vary for adults and children. Studies were excluded if the definition of remission was not reported or unclear. Studies on remission of cancer were excluded as this is already a well-researched area [26]. Registry data and studies that did not assess remission using EHRs were excluded. Studies that reported on remission of symptoms (eg, seizures) rather than remission of an LTC of interest were also excluded. Search limits for studies on humans were applied. No language restrictions were applied.

### Selection of Sources of Evidence

Search results were exported to EndNote (Clarivate) for deduplication and then imported into the Rayyan collaborative systematic review platform for screening and final study selection. Titles and abstracts of records were first screened against eligibility criteria independently by 2 reviewers (BB and HH). Discrepancies in study selection were resolved by discussion between the 2 reviewers. Full texts of potentially relevant records were sought, retrieved, and reviewed in detail by HH.

### Data Charting Process and Data Items

Data were extracted from each eligible study using a structured table developed a priori by HH (following discussions with the research team). Data extracted included the LTCs assessed, author name and publication date, country, study design, population studied, study aims, remission definition (eg, clinical criteria and biomarkers), and key findings related to remission. Data were extracted by HH, and 50% of the extracted data were validated by BB. Where a single study (from the same first author) generated multiple papers, reports, or abstracts, we included the paper with the most comprehensive definition of remission. We did not contact investigators to obtain or confirm information.

### Critical Appraisal of Individual Sources of Evidence

Quality of the identified studies was not assessed as this is not a requirement of scoping reviews.

### Synthesis of Results

A narrative approach was taken to summarize our findings from included studies. The total number of included studies, characteristics of studies (eg, study country, study design, population studied, and setting) was described. Studies were categorized into groups of interrelated conditions, and the number of studies that focused on each specific condition and the characteristics of the studies within each group summarized. Similarities and differences in definitions and methods used to identify remission of LTCs across studies were highlighted.

### Consultation With Clinicians and Data Experts

To ensure the clinical applicability of our findings, the results were discussed with a group of clinicians and data experts. These discussions were pivotal in refining the identified operational definition of remission within EHRs, incorporating insights from real-world clinical practice. Key considerations included the variability in clinical decision-making, the complexities of disease progression, and the practical limitations of current EHR systems. This collaborative process was essential for aligning our findings with the realities of patient care and the capabilities of existing health informatics systems.

We further cross-referenced any remaining conditions from our previously agreed list with expert clinical input to derive a refined list of long-term conditions amenable to remission assessment. This was an iterative process underpinned by clinical judgment to ensure both practical feasibility and clinical relevance. We then searched for relevant medical codes used to define remission (based on our agreed definitions) using Clinical Practice Research Datalink (CPRD) Aurum medical and product data dictionaries. CPRD Aurum contains routinely collected primary care data from practices across England [27]. We also searched coding systems (eg, GitHub repositories, London School of Hygiene and Tropical Medicine Data Compass) and published code lists for any relevant codes to ensure a complete code list. Where older code lists were identified, we used description terms from these code lists to identify relevant codes in our data.

## Results

### Selection of Sources of Evidence

**Figure 1.**
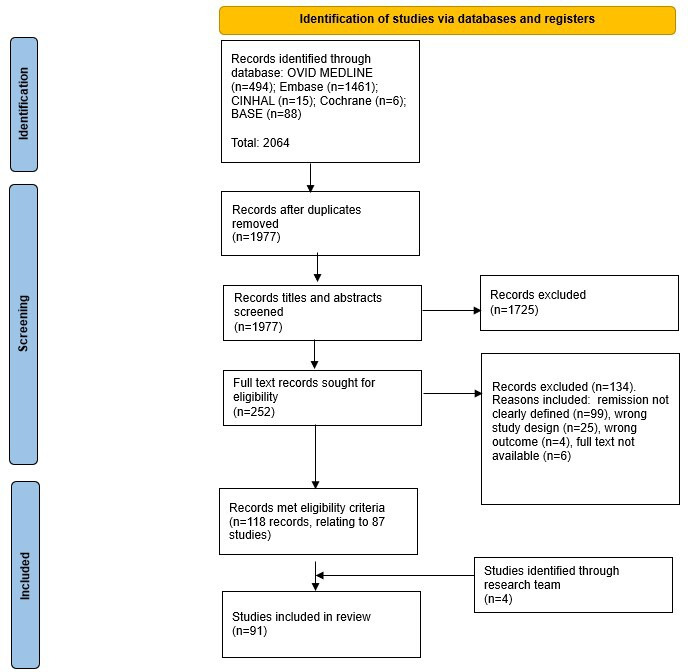
PRISMA (Preferred Reporting Items for Systematic Reviews and Meta-Analyses) flow diagram illustrating search results and screening process.

Figure 1 illustrates the outcomes of the systematic search and selection process. A total of 2064 records were identified through our searches. Following deduplication (n=87/2064, 4.2%) and title or abstract screening, 252 (12.2%) records met the criteria for full-text review. Of those records, 53.2% (134/252) were excluded following full-text review. A total of 91 (36.1%) final studies were included in our review, which included 4 additional studies identified by the research team.

### Characteristics of Sources of Evidence

Included studies were published between 2008 and 2025. The majority of the included studies were conducted in the United States (49/91, 53.8%) [28-76] followed by the United Kingdom (n=13, 14.3%) [13,77-88] and China (n=4, 4.4%) [89-92]. Three (3.3%) studies were conducted in Australia [93-95] and Saudi Arabia [96-98]. Two (2.2%) studies each were conducted in Italy [99,100], Japan [101,102], New Zealand [103,104], Spain [105,106], and Turkey [107,108]. One (1.1%) study each was conducted in Austria [109], Belgium [110], Denmark [111], Finland [112], Kuwait [113], Pakistan [114], South Korea [115], and Sweden [116]. For 1 (1.1%) study, it was unclear which country the study was based [117]. The included studies comprised cohort studies (n=70, 76.9%) [13,28-31,33,35-38,40-42,45,47-51,53-69,71,72,74-79,82-84,86,89-92,95,97,99-106,109-112,114-117], retrospective reviews (n=14, 15.4%) [32,39,43,44,46,52,70,80,81,85,93,98,108,113], cross-sectional studies (n=6, 6.6%) [34,73,88,94,96,107], and an exploratory study (n=1, 1.1%) [87]. Most studies focused on a general adult population (aged ≥18 y) or did not specify an age range, with a small number specifically investigating older populations (eg, veterans or those aged ≥60 y; n=6, 6.6%) [34,35,38,41,81,89] or specific age ranges (n=6, 6.6%) [36,63,75,96,97,112]. Sample sizes ranged from 12 patients to 72.9 million adults. Summary characteristics of the included studies are presented in Textbox 1. Full details of all included studies are available in Supplementary Table 2 in Multimedia Appendix 2.

Textbox 1. **Characteristics of included studies.**

- Number of publications: 91
- Publication date range: 2008‐2025
- Country of study: United States (n=49), United Kingdom (n=13), China (n=4), Australia (n=3), Saudi Arabia (n=3), Italy (n=2), Japan (n=2), New Zealand (n=2), Spain (n=2), Turkey (n=2), Austria (n=1), Belgium (n=1), Denmark (n=1), Finland (n=1), Kuwait (n=1), Pakistan (n=1), South Korea (n=1), Sweden (n=1), and unclear (n=1)
- Study design: cohort studies (n=70), retrospective reviews (n=14), cross-sectional studies (n=6), and experimental or exploratory studies (n=1)
- Population: general or unspecified adult population (n=79), midlife population (n=6), and older populations (age ≥60 y; n=6)
- Sample size range: 12-72.9 million patients
- Condition studied: inflammatory bowel disease (n=41), type 2 diabetes (n=15), depression (n=15), alcohol or drug misuse (n=8), asthma (n=3), multiple sclerosis (n=3), anemia (n=1), chronic kidney disease (n=1), autoimmune pancreatitis (n=1), epilepsy (n=1), heart failure (n=1), hypertension (n=1), and multiple long-term conditions (n=1).

### Critical Appraisal Within Sources of Evidence

The risk of bias in studies was not assessed, in line with scoping review methodology.

### Results of Individual Sources of Evidence: Remission Findings

The majority of the included studies focused on remission in inflammatory bowel disease (IBD; 45.1%, 41/91) [43-61,80-85,90-95,99-101,106,109-111,113,115,117], type 2 diabetes (n=15, 16.5%) [13,62-69,86,87,102-104,114] (one additionally assessing remission of hypertension [103]), and depression (n=15, 16.5%) [32-42,76,78,105,108]. Eight (8.8%) studies assessed remission of drug or alcohol misuse [70-75,107,112], and 3 (3.3%) studies each assessed remission of asthma [29-31] and multiple sclerosis [96-98]. One (1.1%) study each assessed remission in each of anemia [28], chronic kidney disease (CKD) [77], autoimmune pancreatitis [116], epilepsy [79], and heart failure [89]. Additionally, 1 (1.1%) study investigated remission in MLTCs [88].

### Results of Individual Sources of Evidence: Definitions of and Methods Used to Identify Remission in LTCs

#### Overview

**Figure 2.**
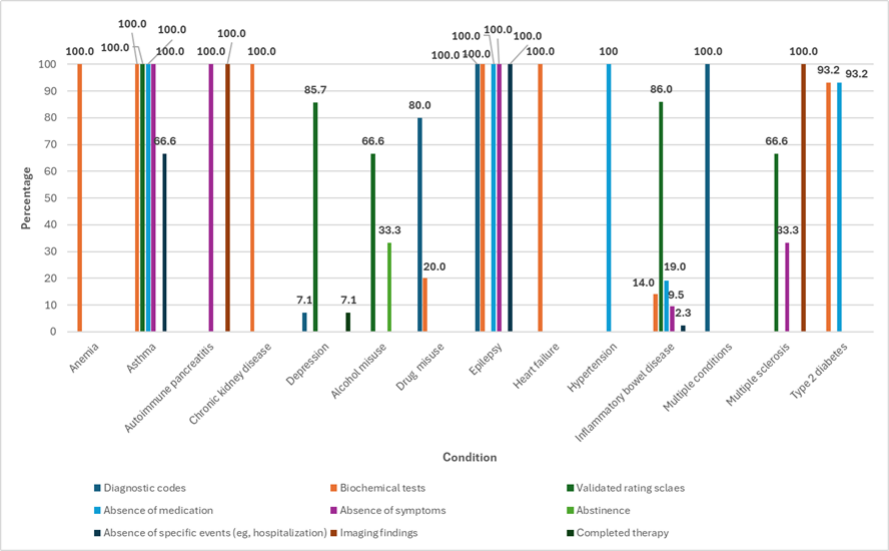
Percentage of studies on specified conditions that used each remission indicator.

Across studies, remission was typically defined using one or more diagnostic codes, validated rating scales, biochemical markers, or the absence of condition-specific events (eg, hospital admissions) following the discontinuation of prescribed pharmacological treatments. Figure 2 presents the distribution of remission indicators across studies for each condition. Most studies defined remission using biochemical tests or markers and validated rating scales.

#### Autoimmune Conditions

##### Inflammatory Bowel Disease

Studies that assessed remission of IBD focused either on Crohn disease (9/41, 22%) [44,50,52,83,95,99,109,111,117] or ulcerative colitis (n=8, 19.5%) [43,55,56,58,59,90,92,115] or both (n=24, 58.5%) [45-49,51,53,54,57,60,61,80-82,84,85,91,93,94,100,101,106,110,113].

**Figure 3.**
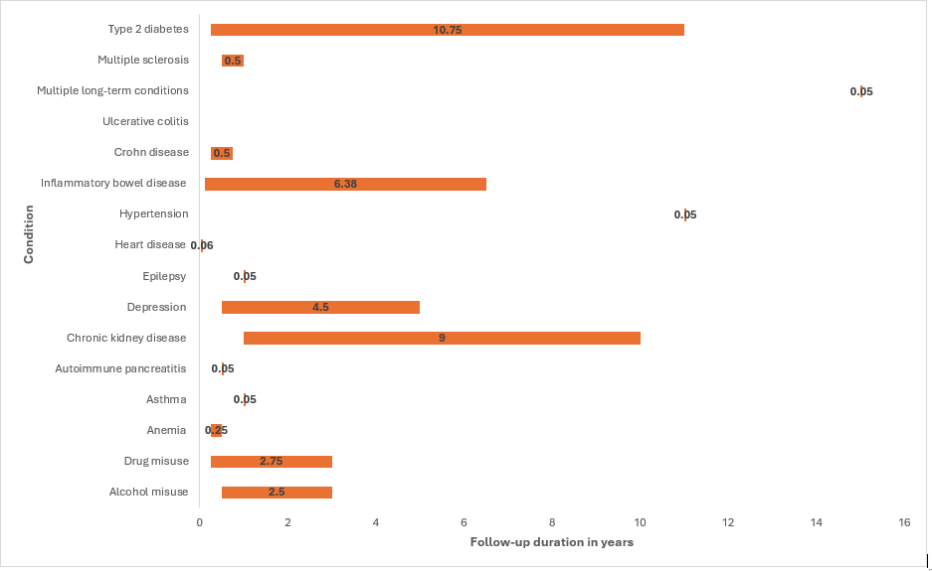
Range of follow-up duration across studies per condition.

All 9 (100%) studies on remission of Crohn disease assessed clinical remission, with 2 (22.2%) specifying steroid-free clinical remission [99,117]. One (11.1%) study each additionally assessed endoscopic remission [82] and biomarker remission [109]. Most studies (n=5, 55.5%) assessed clinical remission of Crohn disease using the Harvey-Bradshaw Index; the majority of studies (n=4, 44.4%) [44,83,95,99] considered a score of <5 at follow-up as remission, while 1 (11.1%) study used a cutoff score of ≤5 [111]. One (11.1%) study defined clinical remission using Crohn Disease Activity Index score of 150. Two (22.2%) studies defined clinical remission based on stool frequency and abdominal pain, with different thresholds [52,109]. One (11.1%) study defined clinical remission as closure of all baseline fistulas [50]. Meade et al [83] additionally defined endoscopic remission using Simple Endoscopic Score for Crohn disease (SES-CD; ≤2 indicating remission). Pokryszka et al [109] additionally assessed biomarker remission based on fecal calprotectin values of ≤150 µg/g [107] and reported lower rates of clinical versus biomarker remission. Length of follow-up varied across studies and ranged from 3 to 6 months (Figure 3).

Seven (87.5%) of 8 studies on ulcerative colitis remission assessed clinical remission [43,56,58,59,90,92,115]. Two (25.0%) studies considered steroid-free clinical remission [56,58]. Clinical remission was mostly defined as a partial Mayo score <2 at follow-up [56,58,59,115]. One (12.5%) study defined clinical remission as partial Mayo score ≤2 without any subscore of >1 [90]. Two (25.0%) studies defined clinical remission using Simple Clinical Colitis Activity Index and reported a cutoff score of <2 [43] or ≤2 [92] at follow-up as clinical remission. Four (50.0%) studies assessed and defined endoscopic remission as Mayo Endoscopic Score (MES) of ≤1 [55,59,92,115], with 1 (12.5%) study considering endoscopic remission as MES of ≤1 or absence of erosions or ulcerations [59]. Zeina et al [55] additionally assessed histologic remission, defined as Simplified Geboes Score ≤0.2. Length of follow-up varied across studies and ranged from 6 weeks to 6.5 years (Figure 3).

Twenty-four (100.0%) studies assessed clinical remission in IBD (both Crohn disease and ulcerative colitis). Three (12.5%) studies specified steroid-free clinical remission [54,81,113]. Studies used multiple scales, including the Harvey-Bradshaw Index, with most (50.0%) defining remission as cutoff score of <5 [45,46,48,54,61,80,81,85,106] or <4 [47,49,93] at follow-up; partial Mayo score of <2 [48,49,51,61,80,84] or ≤2 [93,101] at follow-up; Simple Clinical Colitis Activity Index ≤2 [45,53,54,81] or <2 [49,51,80] at follow-up; Crohn Disease Activity Index <150 [91,101]; the Ulcerative Colitis Activity Index ≤4 [47] at follow-up. Two (8.3%) studies on clinical remission of IBD additionally considered absence of symptoms [46,110] and/or hospitalization [46]. Seven (29.2%) studies assessed endoscopic remission [51,53,57,91,100,110,113], defining remission as MES <2 [51,100,110,113], SES-CD of 0 [53,57], SES-CD of <3 [91], or SES-CD of <4 [100] at follow-up. Six (25.0%) studies assessed biochemical remission [80,82,84,94,100,106], defining remission as fecal calprotectin of <250 µg/g [82,84,94,106] or fecal calprotectin of <150 μg/g [100] at follow-up. Two (8.3%) studies additionally assessed remission as C-reactive protein ≤5 mg/L [84] or C-reactive protein <10 mg/L and/or albumin >35 g/L [82]. Length of follow-up ranged from 8 weeks to 6.5 years (Figure 3).

##### Autoimmune Pancreatitis

One (100.0%) study assessed the outcomes of remission in autoimmune pancreatitis [116]. Remission was defined as the absence of clinical symptoms (asymptomatic, new-onset diabetes, acute pancreatitis, obstructive jaundice, weight loss, and abdominal pain) and absence of pancreatic abnormalities on imaging at 6-month follow-up [116].

### Cardiometabolic Conditions

#### Type 2 Diabetes

Thirteen (86.7%) of the 15 studies assessing remission of type 2 diabetes defined remission as controlled glycemia for a defined period in the absence of antidiabetic medication [13,62-69,86,87,102]. Most (n=11, 73.3%) of these studies assessed glycemic control using glycated hemoglobin A_1c_ biochemical tests, although thresholds varied and included <6.5% (48 mmol/mol) [13,63-69,86,87,102] or <7% (53 mmol/mol) [62]. Duration of glycemic control in absence of medication varied across these studies and included ≥3 months [62,69,87], ≥6 months [13], ≥1 year [63-67,86,102,114], or ≥5 years [68]. One (6.7%) separate study defined type 2 diabetes remission in bariatric and metabolic surgery patients as glycemic control (hemoglobin A_1c_ level<43 mmol/mol [6.1%]) at 6 months [104]. Another study defined type 2 diabetes remission based on the absence of medication at 11 years following gastric bypass surgery [103].

#### Heart Failure

One (100%) study assessed remission of heart failure using blood test results [89]. Heart failure was defined as persistent elevated N-terminal pro-B-type natriuretic peptide levels within 7 and 30 days.

#### Hypertension

Sheikh et al [103] assessed hypertension remission based on the absence of antihypertensive medication. We did not identify other studies that assessed remission of hypertension (rather than control). However, 1 (1/91, 1.1%) of our included studies (that assessed remission in depression) assessed hypertension control using blood pressure measurements at 6-month follow-up [39]. Different cutoffs were used for different age groups (18-59 and 60-85 y) and presence of diabetes.

### Related Conditions

Sheikh et al [103] assessed hypertension remission based on the absence of antihypertensive medication. We did not identify other studies that assessed remission of hypertension (rather than control). However, 1 (1/91, 1.1%) of our included studies (that assessed remission in depression) assessed hypertension control using blood pressure measurements at 6-month follow-up [39]. Different cutoffs were used for different age groups (18-59 and 60-85 y) and presence of diabetes.

#### Anemia

One (100%) study on patients with chronic stable heart failure assessed remission of anemia [28]. Remission of anemia was defined as normalization of hemoglobin levels (≥12 g/dL for men and ≥11 g/dL for women) with at least improvement in ≥0.5 g/dL at 6-month follow-up.

#### Chronic Kidney Disease

One (100%) study on CKD remission defined remission using biochemical tests (estimated glomerular filtration rate ≥60 mL/min/1.73 m^2^) at 1-, 5-, and 10-year follow-up [77].

#### Depression

Remission of depression was mostly defined as normal scores on validated rating scales such as Patient Health Questionnaire-9 scores of <5 [32,34,36,38,39,42,78], ≤5 [33,41], <10 (in patients receiving electroconvulsive therapy) [37], or a 50% decrease from baseline in Patient Health Questionnaire-9 score (in patients aged ≥60 y) [35], Hamilton Depression Rating Scale ≤7 [108], or Geriatric Depression Scale <3 [76] at follow-up. Follow-up varied from 2 months to 1 year after intervention, with most studies assessing remission at 6 months [32,36,39,41] and/or 1 year [33,34,37,78]. One (1/15, 6.7%) study assessed remission using both the Geriatric Depression Scale and remission codes in the EHR over a median follow-up of 53 (IQR 42.1-60.5) months (4.4 y) [76]. Another (6.7%) study assessed remission using codes in the EHR [40]. One (6.7%) study considered remission of major depression as completion of 6 months of therapy [105].

#### Alcohol or Drug Misuse

Eight (100%) studies assessed remission of drug or alcohol misuse [70-75,107,112]. Three (37.5%) studies focused on alcohol misuse [70,71,112]. Two (25%) studies defined remission as negative screening (using the validated alcohol harm assessment tool (Alcohol Use Disorders Identification Test - Consumption score<5) [70] or the National Institute on Alcohol Abuse and Alcoholism single screening question) at follow-up. Follow-up varied across the 2 (25%) studies (1 y [70] and up to 3 y [71]). Rautiainen et al [112] assessed remission of alcohol misuse over a minimum follow-up period of 6 months and defined remission using diagnostic codes or health professional notes in the EHR.

Four (80%) of 5 studies that assessed remission of drug misuse defined remission based on clinical codes (diagnostic codes for remission or absence of codes relating to opioid positivity) [72-75]. Follow-up of studies ranged from 3 months to 3 years.

#### Asthma

Three (100%) studies [29-31] assessed clinical remission of asthma, with 2 (66.7%) reporting a 1-year follow-up [30,31]. For all 3 studies, remission of asthma was defined using multiple clinical indicators. These included the absence of asthma exacerbations, absence of asthma medication, ≥2 normal Asthma Control Test levels, ≥2 normal pulmonary function test values, lung function stabilization, and no absence from work or school due to asthma.

#### Epilepsy

One (100%) study assessed remission in epilepsy at 1-year follow-up [79]. This study defined remission as absence of seizures, absence of antiepileptic drugs, and the absence of all seizure-related health care events, such as general practitioner appointments or hospitalizations.

#### Multiple Sclerosis

Three (100%) [96-98] studies assessed remission of multiple sclerosis, with 2 (66.7%) reporting a follow-up of 1 year [96,97]. Remission was indicated from no evidence of disease activity, which comprised (1) no new or enlarged T2- or gadolinium-enhancing lesions, (2) no clinical relapse (ie, return of old symptoms or worsening of current multiple sclerosis symptoms), and (3) no disability progression (ie, increased walking difficulty leading to progression or an increase in Expanded Disability Status Scale of ≥1.5 between 2 time points).

#### Multiple Long-Term Conditions

One (100%) study assessed remission in 32 LTCs. Identification of remission was based on clinical codes (for remission) available in primary care EHRs [88].

### Synthesis of Results

**Figure 4.**
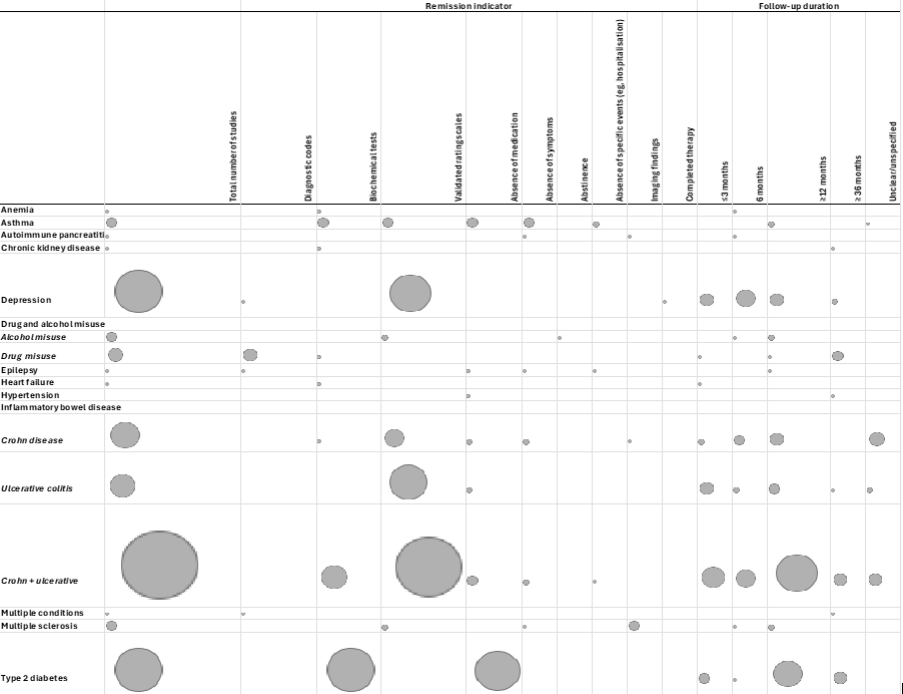
Evidence map of included studies.

Figure 4 presents an evidence map of remission definition across studies included in the review and highlights potential gaps in the literature. Our review highlighted a lack of studies that examined the extent to which varying follow-up periods or the use of different remission indicators (laboratory tests vs diagnostic remission codes) influenced remission rates, except for studies on depression and IBD that reported remission rates across different follow-up durations. Some studies (eg, on IBD) did not specify the absence of medication in their definition of remission, making it less clear whether patients were truly in remission and making cross-study comparisons difficult.

### Operationalization of Remission in EHR Data

This review facilitated the identification of conditions for which remission is measurable within EHRs. Although the scoping review highlighted additional conditions with theoretical potential for remission, discussions with clinicians and CPRD data experts indicated that, in many instances, the lack of suitable EHR-derived metrics or insufficient sample sizes would preclude their meaningful inclusion. Accordingly, we restricted our final list to conditions for which remission was both plausibly measurable and supported by validated indicators within the CPRD dataset, recognizing that this pragmatic approach may omit some conditions capable of achieving remission. Table 1 presents our refined list of long-term conditions based on clinical input and availability of codes in EHRs.

**Table 1.** Recommendations for definition of remission of long-term conditions (in addition to use of diagnostic [remission] codes) based on scoping review findings and discussions with clinicians and data experts.

Long-term condition and explanation of how remission is defined within studies	Period	Clinical considerations
Anemia		
Normalization of hemoglobin levels (≥12 g/dL for men and ≥11 g/dL for women) with at least improvement in ≥0.5 g/dL for 3 mo	≥3 mo	Insufficient information on anemia type or cause hinders meaningful remission assessment
Alcohol dependency		
Screening negative (AUDIT-C^a^ <5) with at least a 2-point reduction from the previous score	≥12 mo	Interpretation should ensure adequate follow-up across participants and account for the possibility that missing data may reflect follow-up occurring in specialist drug and alcohol services, which are not routinely captured in standard health records
Asthma		
No asthma-related event (defined as GP^b^) consultation or admission with a diagnosis code for asthma), no asthma exacerbation, normal lung function and asthma control, and no current use of any asthma medication	≥12 mo	It is important to consider the follow-up period and the age at diagnosis, as some conditions—such as asthma in children—may resolve over time
CKD^c^		
eGFR^d^≥60 mL/min/1.73 m^2^	≥3 mo	More likely indicative of early-stage CKD, given that the condition is traditionally regarded as progressive and irreversible
Depression		
PHQ^e^-9 score<5 (following acute treatment)	≥6 mo	Completion of the PHQ and attendance at the clinic are required; therefore, the absence of a recorded score may reflect factors such as nonattendance or administrative omissions rather than clinical recovery. Furthermore, a 6-mo follow-up period may be insufficient, as conditions such as depression can relapse and remit over the course of several years or even decades
Diabetes		
HbA_1c_^f^ <6.5% without the use of any antidiabetic medication	≥6 mo	No additional comment
Endometriosis		
Symptom (chronic pelvic pain and dysmenorrhea) resolution	≥6 mo	It is also important to consider pregnancy as a potential reason for the absence of diagnostic codes over a period exceeding 6 mo
Epilepsy		
No seizure activity, having no new antiepileptic drug attempts, and the absence of all seizure-related health care events (ie, seizure-related hospitalization or seizure-related GP or outpatient visit), QOF^g^ data, and medical codes indicating a seizure	≥12 mo	No additional comments
Hypertension		
Normal BP^h^ in the absence of antihypertensive drugs. Normal BP varies for age groups: (1) aged 18‐59 y: BP <140/90 mm Hg, (2) aged 60‐85 y with diabetes: BP <140/90 mm Hg, (3) aged 60‐85 y without diabetes: BP <150/90 mm Hg	≥6 mo	Consider follow-up period, and age-related physiological changes can contribute to an increased risk of hypotension with older age
Acute heart failure		
N-terminal pro-b-type natriuretic peptide level persistently decreased above 1.5 times the baseline level	≥6 mo	No additional comments
Inflammatory bowel disease		
*For ulcerative colitis*: a score of ≤3 on the Harvey-Bradshaw Index or ≤4 on the Ulcerative Colitis Activity Index; endoscopic remission (Mayo Endoscopic Score of 0 or 1) and histologic remission (Simplified Geboes Score ≤0.2); ulcerative colitis endoscopic index of severity score=0 at the last follow-up and no use of medication	≥12 mo	No additional comments
*For Crohn:* Harvey-Bradshaw Index or ≤4; FCP^i^ remission rate (FCP <250 µg/g); Crohn Disease Activity Index <150 points and no use of medication	—^j^	No additional comments
Chronic liver disease and alcoholic liver disease		
Absence of symptoms, decrease in serum glutamate oxaloacetate transaminase level to less than twice normal and other standard liver function (serum gamma globulin and bilirubin levels) to normal and disappearance from the liver biopsy of established features of disease activity	≥6 mo	No additional comments
Thyroid disease		
Relief from symptoms, and either of the following: thyroid-stimulating hormone levels in the reference range (also known as the “normal range”: 1‐2 MIU/L) OR free thyroxine (Free T4) levels in the reference range: 77.2‐1543.1 nmol/L OR free triiodothyronine (Free T3) levels in the reference range: 5‐7 pmol/L	≥12 mo	Preference for clinical biomarkers over relief of symptoms

a AUDIT-C: Alcohol Use Disorders Identification Test - Consumption.

b GP: general practitioner.

c CKD: chronic kidney disease.

d eGFR: estimated glomerular filtration rate.

e PHQ: Patient Health Questionnaire.

f HbA_1c_: hemoglobin A_1c_.

g QOF: Quality and Outcomes Framework

h BP: blood pressure.

i FCP: fecal calprotectin.

j Not applicable.

## Discussion

### Summary of Evidence

This scoping review synthesized evidence on how remission of LTCs has been defined and operationalized within EHRs. We identified 91 studies [13,27-117] covering 12 LTCs, including type 2 diabetes [13,62-69,86,87,102-104,114], depression [32-42,76,78,105,108], IBD [43-61,80-85,90-95,99-101,106,109-111,113,115,117], anemia [28], epilepsy [79], CKD [77], autoimmune pancreatitis [116], alcohol and/or drug misuse [70-75,107,112], heart disease [89], asthma [29-31], hypertension [103], and multiple sclerosis [96-98]. The review improved our understanding of how remission can be identified and operationalized in EHRs as criteria used to define and identify remission in studies included diagnostic (remission) codes, validated symptom scales, biochemical markers, absence of condition-specific events, and discontinuation of treatment [13,27-117]. However, criteria and follow-up periods differed widely across studies, even within the same condition (Figures 2-4), highlighting a lack of standardized definitions for remission. Using our scoping review findings and discussions with clinicians and data experts, we propose standardized definitions for remission of LTCs that will improve identification in EHRs (Table 1).

Overall, our review indicates that remission of LTCs remains relatively underresearched as many relevant studies identified in our searches did not clearly define remission (Figure 1). Most studies were conducted in the United States [28-76] or the United Kingdom [13,77-88] and focused on IBD [43-61,80-85,90-95,99-101,106,109-111,113,115,117] and diabetes [13,62-69,86,87,102-104,114]. The lack of studies in some countries likely reflects differences in the extent of adoption of EHRs for clinical care, particularly for low-income countries where EHR adoption may be at its earlier stages [118]. These differences may also capture differences in clinician coding, with some studies indicating clinicians in the United States, for example, spend more time actively using the EHR [119]. Furthermore, we did not identify any studies on remission of some conditions known (and deemed by clinicians we consulted) to be amenable to remission, such as peptic ulcer disease, chronic urinary tract infection, chronic liver disease, and alcoholic liver disease, peripheral vascular disease, transient ischemic attack, stroke, tuberculosis, and thyroid disease (standardized definitions provided in Table 1). Our finding that the majority of studies described remission of certain conditions may reflect differences in the clinical focus of health conditions, research priorities, and policies across countries [118,120]. For example, in the United Kingdom, the Quality and Outcomes Framework—a pay-for-performance scheme—is associated with better recording and monitoring of certain long-term conditions (such as diabetes and asthma) in clinical practice [120].

Across studies, variation in remission criteria for the same condition may partly be explained by differences in health systems, as well as differences in the availability of remission information [118,121]. For example, settings with fragmented health systems may not reliably include criteria such as absence of hospitalization in their definition of asthma remission, whereas this would be possible for integrated systems with linked primary and secondary care data [121]. Diagnostic remission codes were used for only a few of the conditions, as may be expected due to a lack of remission codes for many conditions [88]. Remission codes, where they exist, may not be commonly used due to clinician coding burden and uncertainty [122,123]. Thus, it is important to include a range of criteria in the definition of remission to allow more accurate estimates of remission. Follow-up duration for remission also varied for studies, which may impact estimates of remission rates [124]. A better understanding of how remission rates vary over time (and any factors affecting remission over time) could improve understanding of observation periods required for remission definitions.

### Comparison With Existing Literature

Previous reviews on remission have examined single conditions (eg, asthma [125,126], psoriasis [127], diabetes [128,129], rheumatoid arthritis [130], psychosis [131], and systemic lupus erythematosus [132]) and did not focus on EHRs. Our findings are consistent with previous studies [125-127] in that we observed varying definitions of remission of each LTC. Moreover, these previous studies identified that individuals with MLTCs were less likely to achieve remission of specific conditions [125,127]; however, the studies did not assess remission of comorbidities [125-127]. Our review offers an opportunity to advance understanding of remission of long-term conditions by providing consensus-driven definitions of remission of multiple LTCs [Table 1]. This will allow studies (including those focused on single conditions) to explore and consider the impact of remission in other comorbidities in a consistent, rigorous way that can allow cross-study comparisons. Unlike these previous reviews on remission, our study focuses on EHRs and presents practical approaches to define remission in real-world settings where barriers such as variation in coding, fragmented health care, and health system factors make it challenging to study remission [118-123]. Our review extends previous work on remission in MLTCs [88] by presenting a comprehensive approach to defining remission in EHRs beyond the use of remission and resolved codes to better identify remission and in turn improves population-based research and clinical outcomes of LTCs.

### Implications for Research and Clinical Practice

Studies on LTCs using EHRs, including those assessing prevalence and trajectories, often do not account for remission of conditions [88], which may have implications for our understanding of MLTC [133-135]. This may be partly due to a lack of consensus for identifying remission, as identified by this review. We combined findings from our review with discussions with a clinical team, data experts, and a review of existing codes for remission in EHRs. This comprehensive approach can be used to advance research in this area and improve understanding of remission, particularly in the context of MLTC [136]. Future work may validate whether remission, as identified in EHRs, reflects true remission rates [137]. As with all EHR-based studies, there is bias due to incomplete data or missing information from subgroups [138]. Future work will need to consider missing data handling techniques to address this in order to ensure remission rates are not overestimated (eg, due to patients not consulting or being tested). Further research on remission may improve understanding of which conditions are more likely to achieve remission in patients living with MLTC, as well as factors and outcomes associated with remission. This understanding can improve the evidence base on remission and allow clinicians and patients to work toward remission, as well as improve care and outcomes of MLTC [139]. Furthermore, the range of criteria used to define remission of LTCs (Figure 2) highlights a need for a comprehensive approach to clinical coding to allow accurate identification and monitoring of remission. Further research is needed to assess the reliability of EHRs in capturing remission and to explore the wider factors associated with the documentation of remission in health records.

### Strengths and Limitations

This study methodology [20] offers a comprehensive and systematic examination of how the remission of LTCs has been defined and operationalized within EHRs. A key strength lies in our integrative approach, which combined evidence from the literature with expert consultation and real-world coding frameworks. This methodological triangulation enhances the relevance and applicability of our findings [136], particularly in the context of MLTCs, where the concept of remission may inform future intervention design and policy development. However, several limitations should be acknowledged. First, although we conducted thorough electronic and gray literature searches, it is possible that some relevant studies, particularly those not using MeSH terms related to EHR, may not have been captured. Additionally, studies involving pediatric populations, cancers, or LTCs outside our predefined list for MLTCs were excluded. This decision was informed by input from our public advisory group, who emphasized the need to focus on noncancer conditions due to the relative lack of understanding around their remission. Nonetheless, this narrowed scope limits the generalizability of our findings to other potentially resolvable conditions [140], and further research will be necessary to develop comparable frameworks for these excluded areas.

Second, for some LTCs, only a small number of eligible studies were identified (n≤1), resulting in limited evidence on which to base definitions of remission. As a result, our synthesis may reflect early or preliminary interpretations for these conditions rather than consensus-based definitions. Similarly, different study characteristics, as well as varying remission indicators and duration of follow-up, made it difficult to directly compare study findings [140]. There was a lack of studies comparing whether remission rates varied based on the choice of remission indicators. Finally, our operational framework for identifying remission was derived largely from coding structures available in the UK primary care and linked datasets. Conditions for which complete or long-term follow-up is less feasible in primary care were not included. As such, the applicability of our findings may vary in international contexts or within health care systems using different data architectures [140]. Future efforts should aim to validate and refine this framework across diverse settings and data environments to enhance its robustness and global utility.

### Conclusions

This review demonstrates that the remission of multiple LTCs can be identified and operationalized using EHRs, although approaches to defining remission and the duration of follow-up varied considerably across studies. While several LTCs have been studied, many others with the potential for remission remain underrepresented in the literature. Our review is innovative, as it uses both evidence synthesis and consultation with clinical and data experts to propose practical approaches to define and implement remission in EHR-based research. The review offers standardized definitions for multiple LTCs. These findings lay the groundwork for improving the identification of remission, particularly in the context of MLTCs, where a better understanding of disease trajectories is critical. Due to variation in clinical coding and missing data (eg, due to lack of test results) [137,138], it is necessary to adopt a comprehensive approach and include all these indicators (as appropriate for the specified condition) to reliably capture remission and evaluate outcomes for patients. Future research may also report on how the choice of remission indicators (eg, diagnostic codes vs biochemical biomarkers) influences findings. Further research may apply, compare, and evaluate standardized definitions across different data sources to assess generalizability and further improve our understanding of the remission of LTCs.

## Supplementary material

10.2196/80796 Multimedia Appendix 1 List of 56 conditions on which our study was based and full details of included studies.

10.2196/80796 Multimedia Appendix 2 Search strategies.

10.2196/80796 Checklist 1 PRISMA-ScR fillable checklist.

## Abbreviations:

CKD: chronic kidney disease
CPRD: Clinical Practice Research Database
EHR: electronic health record
IBD: inflammatory bowel disease
LTC: long-term condition
MES: Mayo Endoscopic Score
MLTC: multiple long-term condition
PRISMA-ScR: Preferred Reporting Items for Systematic Reviews and Meta-Analyses extension for Scoping Reviews
SES-CD: Simple Endoscopic Score for Crohn Disease
