# Supplementary material for: Understanding Remission of Long-Term Conditions Through Electronic Health Records: Scoping Review
Source: J Med Internet Res. 2026 May 19;28:e80796. doi: 10.2196/80796 (PMC13186534; doi:10.2196/80796)
Supplement: Multimedia Appendix 1 [file jmir-v28-e80796-s001.pdf]

Supplementary material

Supplementary Table 1. List of 56 conditions on which our study was based. Conditions with an asterisk (*) are those considered (by the research team following discussions with clinicians) amenable to remission.

**Condition**
Addison disease
Pernicious Anaemia
Anxiety
Aortic aneurysm
Arrhythmia
Asthma
Autism
Bipolar disorder
Bronchiectasis
Solid organ and haematological cancers
Coronary heart disease
Chromosomal abnormalities
Chronic kidney disease
Chronic Lyme disease
Congenital heart disease
Chronic obstructive pulmonary disease
Post-acute COVID-19
Connective tissue disease
Cystic fibrosis
Drug and alcohol misuse
Dementia
Depression
Diabetes mellitus
Eating disorder*
Endometriosis*
Epilepsy*
Gout*
Hearing impairment*
Heart failure*
HIV/AIDS
Heart valve disorder
Hypertension*
Inflammatory bowel disease*
Chronic liver disease and alcoholic liver disease*
Meniere's disease*
Metastatic cancer*
Multiple sclerosis*
Long-term musculoskeletal problems due to injury*
Osteoarthritis

Osteoporosis
Chronic pain
Chronic pancreatitis*
Paralysis
Parkinson's disease
Peripheral neuropathy*
Post traumatic stress disorder*
Peptic ulcer disease*
Peripheral vascular disease
Schizophrenia
Stroke
Tuberculosis*
Thyroid disease*
Transient ischaemic attack*
Chronic urinary tract infection*
Visual impairment*
Venous thromboembolic disease

Supplementary Table 2: Characteristics of included studies

Ref number	Condition	Author	Country	Study design	Population studied	Study aims	How remission/resolution was defined	Key findings related to identification and definition
27	Anaemia	Tang et al., 2008	United States	Cohort study	6159 outpatients with chronic stable heart failure	To determine the characteristics and long-term prognosis of anaemia in ambulatory patients with chronic heart failure	Normalization of Hb levels (≥12 g/dl for men and ≥11 g/dl for women) with at least improvement in ≥0.5 g/dl at 3 & 6 month follow-up in those who presented with anaemia at baseline	43% patients with anaemia at baseline had resolution of their haemoglobin levels. Higher total mortality rates were evident in patients with persistent anaemia (58% vs. 31%, p < 0.0001) or with incident anaemia (45% vs. 31%, p < 0.0001) compared with those with without anaemia at 6 months.
28	Asthma	Howarth et al., 2024	United States	Retrospective cohort study	Asthma patients (18+ years)	To evaluate the extent to which components of CR measure are documented and achieved in asthma patients initiated on respiratory biologics	Clinical remission defined base on the following criteria: no asthma exacerbations, no systemic corticosteroid use, no controller and rescue medication use, ≥2 stable asthma control tests, ≥2 stable pulmonary function tests, and no missed work or school due to asthma	94.2% had documentation for at least one clinical remission (CR) criterion. Among patients with available data, 91.2% met at least 1 criterion, 65.5% met 2+, 28.3% met 3+ and only 6.2% met 4+ criteria in the broader respiratory biologic cohort.

29	Asthma	Carstens et al., 204	United States	Retrospective cohort study	Patients ≥12 years of age (mean age: 55 years) with ≥2 benralizumab prescriptions	To describe real-world outcomes, derived from asthma remission components, in benralizumab-treated patients	Remission at 12 months post treatment initiation defined as: zero exacerbations, no maintenance oral corticosteroid (mOCS) use, and lung function stabilization (FEV1 decrease ≤10% from baseline)	The number of patients (2.1%) who reported FEV1 results during baseline and follow-up was low. The proportion achieving both zero exacerbations + no mOCS use was 41.1% (932/2,247).
30	Asthma	Lim et al., 2024	United States	Retrospective cohort study	707 adult asthma patients (>=18 years) initiated on respiratory biologics (mepolizumab, dupilumab, benralizumab, omalizumab, reslizumab, tezepelumab)	To evaluate the extent to which clinical remission (CR) endpoints are measured in real-world settings in patients with asthma over a 1-year follow-up period after initiating respiratory biologics	Remission definition: No asthma exacerbations, no systemic corticosteroid use (SCS), controller, and rescue medication usage, >=2 stable asthma control tests (ACT), >=2 stable pulmonary function tests (PFT), and no missed work or school due to asthma at 1 year follow-up	The findings of this study reveal the infrequent capture of the different components of CR endpoint in routine practice, thereby limiting the comprehensive evaluation of CR.
114	Autoimmune pancreatitis	Nikolic et al., 2021	Sweden	Retrospective cohort study	12 patients with autoimmune pancreatitis (AIP) treated with rituximab (RTX)	To determine the clinical and imaging response to RTX and summarize the existing data on RTX therapy in patients with AIP type 1 in the literature	Complete remission was defined as the complete absence of both clinical symptoms (asymptomatic, new-onset diabetes, acute pancreatitis, obstructive jaundice, weight loss, abdominal pain) and imaging findings (on magnetic resonance imaging)) 6 months	Twelve (11.7%) of 103 patients with AIP type 1 were treated with RTX during the study period: eight (66.7%) achieved complete and four (33.3%) partial remission

							after initiation of treatment	
75	Chronic kidney disease	Taal et al. (2024)	United Kingdom	Cohort study	1725 patients (median age: 74 years) with confirmed CKD stage 3	To assess the impact on outcomes of fluctuations in CKD diagnostic criteria.	eGFR ≥60 ml/min/1.73 m2 and UACR <3 mg/mmol at each time point (year 1, 5, 10)	Prevalence of remission was 495/1737 (28.5%) at baseline, 424/1618 (26.2%) at year 1, 335/1236 (27.1%) at year 5 and 176/1190 (14.8%) at year 10. Remission was present at all three visits in 15.4%, two visits in 13.0% and one visit in 15.8%. Evidence of remission at 1, 2 or 3 study visits was associated with progressively lower incidence of persistent CKD, CKD progression and death at 10 years

103	Depression	Sicras-Mainar et al., 2010	Spain	Retrospective Cohort study	965 patients >20 years old with major depression	To determine the clinical consequences and economic impac t of using escitalopram (ESC) vs. citalopram (CIT) and venlafaxine (VEN) in patients who initiate treatment for a new episode of major depression (MD)	Patients were considered to be in remission if they completed 6 months of therapy. Random sampling was done to compare the administrative definition of ‘remission’ to whether the remission of major depression was documented in the electronic clinical history	ESC-treated patients achieved higher remission rates compared to CIT (58.0% vs. 38.3%) or VEN patients (32.4%), $p < 0.001$
31	Depression	Angstman et al., 2013	United States	Retrospective review	1550 adult patients (aged > 18 years) from primary care practices, representing all body mass index (BMI) categories, who had a Patient Health Questionnaire (PHQ-9) score of ≥ 10 with follow-up data (PHQ-9 score and weight) at 6 months	To determine whether enrolment in collaborative care management (CCM) for treatment of major depression would have a significant impact on 6-month changes in weight compared with patients treated by their primary care provider with usual care	(PHQ-9 score < 5) at 6 months	Either enrolment in CCM(P = 0.306) nor clinical remission (P = 0.828) was associated with a significant weight gain

32	Depression	Sacks et al., 2014	United States	Prospective cohort study	5253 patients (18+) with depression	To examine the relationship between patient activation, a measure of individuals′ knowledge, skill, and confidence for managing their health, and rates of depression remission and response among patients with depression	Depression remission was defined as having a patient health questionnaire (PHQ-9) score in the follow up year of less than or equal to 5.	Higher patient activation predicted better depression outcomes
33	Depression	Pfoh et al., 2015	United States	Cross-sectional study	Medicare beneficiaries aged 65+ with at least one primary care visit between September 2010 and August 2012	To evaluate conformance to depression screening, management, and outcome quality indicators and to evaluate individual characteristics associated with conformance to these indicators	PHQ-9 score of less than 5 at 12 months	Twenty-three (77%) of the 30 met the quality measure criterion of a subsequent depression scoreless than 5 within 12 months of the initial PHQ-9 score greater than 10
34	Depression	Eguchi et al., 2019	United States	Retrospective cohort study	57 patients (>=60 years old) with depression	To assess if Problem Adaptation Therapy (PATH) worked equally well in English and Spanish speakers.	Remission defined by 50% decrease in PHQ9 score from initial to final visit (end of treatment)	PATH achieved 37% remission in this clinically and culturally complex population

35	Depression	Yates Coley et al., 2020	United States	Cohort study	New episodes (n=5554) of people (aged 13-≥65) receiving psychotherapy for depression between February 2016 and January 2017	To compare measures and examine the relationship between baseline symptom severity and treatment success.	Remission was defined as (PHQ-9 score<5) from 14-180 days after treatment initiation	Remission was attained by 22% and was associated with lower baseline scores
106	Depression	Gurel et al., 2021	Turkey	Retrospective review	107 patients (18+) receiving bi-temporal electroconvulsive therapy (ECT)	To compare the effectiveness of ECT in bipolar disorder (BDD) and major depressive disorder (MDD)	Final (at end of Electroconvulsive therapy) Hamilton Depression Rating Scale (HAM-D) score of ≤7	84% of patient in the BDD group achieved remission, 63% in the MDD group. The total number of ECT sessions during the examined course of ECT in participants who achieved remission did not differ between the BDD and the MDD groups
76	Depression	De Giorgi et al., 2023	United Kingdo m	Cohort study	673,177 patients aged 18-100 years, registered between January 1998 and August 2020, diagnosed with a new episode of depression, and commencing an antidepressant	To assess the real-world acceptability, tolerability, and efficacy of concomitant antidepressant and statin treatment in depression	Patient Health Questionnaire (PHQ)-9 score<5 at 2, 6, and 12 months	Efficacy did not differ between treatment groups (range aOR2-12 months 1.00 and 1.02 for response and remission, range aOR2-12 months − 0.01 and − 0.02 for change in depression score).
36	Depression	Li et al., 2023	United States	Retrospective cohort study	495 patients who received 2 or more electroconvulsive therapy(ECT) treatments	To examine how anaesthetic choice between etomidate and methohexital is associated with real-world clinical outcomes	Remission defined as a PHQ-9 score <10 as assessed closest to 12 months after the initial ECT treatment completion	No significant difference on depression remission, emergency department visits, or hospitalizations 12-months posttreatment.

37	Depression	Liebmann et al., 2023	United States	Cohort study	veteran population (n=48,886)	To compare PHQ-9 scores and rates of remission and response based on the data included in VA's EHR with independent estimates based on survey methods from the VOA.	Patient Health Questionnaire (PHQ)-9 score<5 at 3 months	Aggregated rates of response and remission from EHR data were significantly different from estimates based on representative VOA data. The findings suggest that until patient-reported outcome from EHRs are available for a substantial majority of patients receiving care, aggregated measures of patient outcomes derived from these data cannot be assumed to be representative of the outcomes for the overall population,
116	Depression	Keins et al., 2023	USA	Cohort study	1243 adult (age ≥18), presenting between January 2006 and December 2018 and diagnosed with spontaneous Intracerebral haemorrhage (ICH)	To determine whether depressive symptoms after ICH are associated with inadequate BP control	Clinical remission of depressive symptoms was considered upon meeting both of the following criteria: (1) documented evidence in medical records or billing information of remission of depressive symptoms; and (2) 4-item version of the Geriatric Depression Scale score dropping below 3 at follow-up	Resolution of depressive symptoms was associated with subsequent decrease in systolic (−5.9 mm Hg, SE, 1.4 mm Hg, *P*=0.031) and diastolic (−3.4 mm Hg, SE, 1.1 mm Hg, *P*=0.041) BP measurements., over a median follow-up of 52.8 months

38	Depression	Whiting et al., 2023	United States	Retrospective review	114438 patients from 2 Mayo Clinic family medicine primary care clinics	To assess for differences in patient care outcomes in the primary care setting for patients assigned to an independent practice panel (IPP) or a shared practice panel (SPP)	Depression remission: a score of less than 5 at 6 months (within ±60 days) after an initial PHQ-9 or PHQ-9M score of greater than 9. Hypertension control:(1) aged 18-59 years: BP less than 140/90 mm Hg; (2) aged 60-85 years with diabetes: BP less than 140/90 mm Hg; and (3) aged 60-85 years without diabetes: BP less than 150/90 mm Hg	The IPP clinicians showed improved quality metrics compared with the SPP clinicians for the percentage of assigned patients achieving depression remission (16.6% vs 11.1%; P<.01). This study shows a considerable improvement in depression remission among IPP panels and in cervical cancer screening rates among SPP panels
39	Depression	Deng et al., 2024	United States	Retrospective cohort study	78673 patients (mean age: 55 years)	To analyse depression remission among irritable bowel syndrome patients	Depression remission was defined as 'major depressive disorder, single episode, in full remission' (F32.5), 'major depressive disorder, single episode, in partial remission' (F32.4), and 'major depressive disorder, recurrent, in remission' (F33.4)	Those using antidepressants showed significantly higher rates of depressive remission compared to non-users: risk difference (RD), -0.056; risk ratio (RR), 0.380; and hazard ratio (HR), 0.413.

40	Depression	Pfeiffer et al., 2024	United States	Retrospective cohort study	215 veterans	To characterize clinical outcomes of repeated infusions in routine clinical practice and the frequency and number of infusions used to sustain symptom improvement	PHQ-9 score ≤ 5 at 3 and 6 months	15% had PHQ-9 score ≤ 5. While only a minority of patients treated with IV ketamine for depression experienced response or remission, symptom improvements achieved within the first 6 weeks were sustained over at least 6 months with decreasing infusion frequency
41	Depression	Benster et al., 2025	United States	Retrospective cohort study	232 patients with treatment resistant depression (mean age: 54.5 years)	To predict depressive symptom response and remission following repetitive transcranial magnetic stimulation	(PHQ-9 < 5) repetitive transcranial magnetic stimulation (rTMS)	Patients with comorbid anxiety, obesity, concurrent benzodiazepine or antipsychotic use, and more chronic TRD were less likely to respond or remit following repetitive transcranial magnetic stimulation

77	Epilepsy	Powell et al., 2019	United Kingdom	Cohort study	4388 (adults n=3861; children n=527) newly diagnosed with epilepsy	To assess the evolution of antiepileptic drug (AED) treatment patterns and seizure outcomes in England from 2003 to 2016.	One-year remission was defined as having no new antiepileptic drug(AED) attempts, and the absence of all seizure-related healthcare events (i.e., seizure-related hospitalisation or seizure-related GP or outpatient visit; for instance, a GP visit with a diagnosis recorded as '1B64.00—had a convulsion'), QOF data and Read codes (online supplementary table S2) indicating a seizure at any time for at least 1 year.	Rates of 1-year remission within 2 years of starting treatment increased in adults (era 1: 71.9%; era 3: 81.4%) and elderly (era 1: 76.1%; era 3: 81.7%). Overall, 55.5% of patients relapsed after achieving 1-year remission
87	Heart failure	Liu et al., 2023	China	Cohort study	308 patients (Aged 65+ years) diagnosed with acute heart failure coexisting with oliguria	To examine the efficacy of adding tolvaptan (TLV) on improving the prognosis in elderly patients with AHF coexisting with oliguria.	NT-proBNP level persistently decreased above 1.5 times the baseline level within 7 and 30 days	The addition of TLV was clinically effective in increasing urine output, and had favourable effects on alleviating AHF progression and may reduce the risk of all-cause mortality at 7 and 90-day in elderly patients with AHF with oliguria, and TLV had a good safety profile

42	IBD	Blonski et al., 2011	United States	Retrospective review	184 patients with ulcerative colitis (UC)	To compare and contrast the clinical and endoscopic disease activity in UC patients in clinical remission and those with disease activity at the time of their surveillance colonoscopy.	Clinical remission: Simple Clinical Colitis Activity Index (SCCAI)CCAI < 2 and mucosal healing was defined as FES 0 or 1 at time of colonoscopy	Clinical remission is predictive of endoscopic remission and clinical disease activity is predictive of endoscopic disease activity in patients with ulcerative colitis at the time of surveillance colonoscopy
43	IBD	Kane et al., 2013	United States	Retrospective review	56 Crohn disease patients	To evaluate effectiveness of split-dose certolizumab pegol for the treatment of Crohn's disease	Remission was defined by evidence of inactive disease on imaging or histology, no steroid therapy at assessment, a Harvey Bradshaw Index (HBI) <5, or physician assessment of clinical remission if HBI not available.	Sixteen patients (29%) achieved clinical remission, with a median time to remission from dose splitting of 8 months (range, 3-14).
44	IBD	Colman et al., 2014	United States	Retrospective cohort study	88 methotrexate (MTX)- prescribed patients with inflammatory bowel disease	To compare efficacy of low-dose and high-dose MTX treatment regiments among patients prescribed MTX and a biologic medication	Clinical remission: Harvey-Bradshaw index (HBI) <=4, simple clinical colitis activity index (SCCAI) <=2 or by clinical report at 6 months intervals (and up to 42 months)	When combined with anti-TNF therapy, MTX prescribed at doses of >12.5 mg/week were more effective at maintaining clinical remission than lower doses, but did not affect overall steroid use, anti-TNF dosing, surgery or endoscopic inflammation

109	IBD	Saleem et al., 2014	Denmark	Retrospective cohort study	33 patients with Crohn's disease	To evaluate measurement of Faecal calprotectin (FCALP) at regular intervals before and during therapy for IBD	Clinical remission: Harvey Bradshaw index score <5 at 4- and 12-months post therapy	FCALP can be used to monitor response to anti- TNF therapy in Crohn's disease and provides an objective marker of inflammation.
45	IBD	Merkely et al., 2015	United States	Retrospective review	24 patients with IBD who received intravenous immunoglobulin between February 2011-June 2013	To investigate the use of intravenous immunoglobulin (IVIg) to manage patients with IBD who are refractory or have contraindications to standard therapies	Clinical remission was defined as Harvey-Bradshaw Index score <5, no hospitalizations or surgeries after IVIg, or symptom resolution at 16 months follow-up	3 (12.5%) obtained remission with IVIg. IVIg is safe and effective in the short-term management of patients with IBD when standard therapies are contraindicated.
78	IBD	Bhandare et al., 2019	United Kingdom	Retrospective review	96 IBD patients	To assess to effect of switching from originator infliximab IFX-O infliximab biosimilar (CT-P13)	Biochemical remission; clinical remission (pMayo < 2 and HBI < 5) at 13 months	Clinical efficacy and loss of response rates with CT-P13 appears to be similar to IFX-O.

46	IBD	Ahsan et al., 2020	United States	Cohort study	1259 Inflammatory Bowel disease (IBD) patients (median age: 40 years)	To characterize differences in IBD treatment regimens among patients with high and low sugar consumption and if medication patterns exist within subgroups that are in remission.	Remission was defined as a score of ≤ 3 on the Harvey-Bradshaw index or ≤4 on the Ulcerative Colitis Activity Index.	High sugar consumption was associated with increased anti-TNF medication use, higher combination therapy use, and lower 5-ASA use. Among the subgroup of high sugar consumers, those not in remission used more adalimumab and steroids
79	IBD	Ibraheim et al., 2020	United Kingdo m	Retrospective review	48 patients (aged 60+ years)	To determine the clinical effectiveness and safety of vedolizumab in older IBD patients within a real-world multicentre UK cohort	Clinical remission (Harvey Bradshaw Index [HBI]<5 or Simple Clinical Colitis Activity Index [SCCAI]<3) and corticosteroid-free remission (HBI<5 or SCCAI<3 without concomitant steroids) at week 14	Rates of clinical response, remission and corticosteroid-free remission at week 14 were 64%, 48% and 30%, respectively. By week 52, the rates of clinical response, remission, and corticosteroid-free remission were 52%, 38%, and 32%, respectively

108	IBD	Jorissen et al, 2021	Belgium	Retrospective cohort study	142 inflammatory bowel disease (IBD) patients on thiopurines abov e the age of 60	To assess the long-term outcome of elderly IBD patients after discontinuation of thiopurine while in clinical remission	Clinical remission: physician global assessment as a stable stool frequency without blood and absence of abdominal pain. Endoscopic remission was defined as a MAYO endoscopic sub score of 0 or 1 (UC) and simple endoscopic score for Crohn's disease (SES-CD) <3 or absence of ulcerations in case no SES-CD was available (CD) over a median follow-up of 55 months	All 91 patients were in clinical remission at the time of discontinuation of TP and had normal laboratory markers (C-reactive protein < 5.0 mg/L). Thirty-seven patients (40.7%) had an endoscopy performed at the time of discontinuation and also had endoscopic remission. In total, 63 patients (69.2%) remained in clinical and/or endoscopic remission during a median follow-up of 55 months (IQR 33–90 months)
47	IBD	Vachon et al., 2020	United States	Retrospective cohort study	163 individuals >=18 years old with a diagnosis of IBD	To measure the association between IBD-related symptoms and depression over time.	Clinical remission was defined as baseline active disease with subsequent reduction in scores to HBI <= 4 or PMS <= 1 at follow up.	After adjusting for history of depression, clinical response and remission were associated with significant odds of decreased depression score in CD. In UC, this association was not observed.
48	IBD	Fernandez-Cano et al., 2021	United States	Retrospective cohort study	40 patients (with ulcerative colitis or Crohn's disease) (median age 53 years)	To assess effect of vedolizumab in real practice	Clinical remission: Harvey-Bradshaw Index (HBI)<4 or Partial Mayo Index (PMS)<2 at weeks 12,24 or 54	No significant differences in clinical response/ remission at weeks 12, 24 or 52. There were no significant differences in clinical response/remission with the use of vedolizumab.

49	IBD	Lee et al., 2021	United States	Retrospective cohort study	31 Crohn's disease patients (age range: 21-63, median: 38)	To characterize magnetic resonance imaging (MRI) outcomes of perianal fistula at a specialist inflammatory bowel disease (IBD) unit	Clinical remission was defined as closure of all baseline fistulas, occurring at any time during the period of follow-up. Closure defined as absence of any discharge with gentle finger compression on examination	Clinical remission was achieved in 22 of 44 (50%) of patients, 13 of 22 (60%) while on infliximab and 9 of 22 (40%) while on adalimumab.
88	IBD	Xie et al., 2021	China	Retrospective cohort study	372 ulcerative colitis patients (mean age: 41.9)	To analyse the levels of Systemic immune-inflammation index (SII) in ulcerative colitis patients and assess the relationship between the SII and disease activity	Clinical remission: Mayo score <2	Higher SII levels were observed in moderate and severe UC subgroups compared to mild or remission subgroups
113	IBD	Ye et al., 2021	Korea	Retrospective cohort study	105 ulcerative colitis patients (age 19+ years, mean age: 45.3 years)	To examine the real-world effectiveness and safety outcomes of vedolizumab in ulcerative colitis (UC) patients who had failed anti-tumour necrosis factor (anti-TNF) therapy	Clinical remission was defined as a partial Mayo score of at most 2, without any sub score being greater than 1. Endoscopic remission was defined as a Mayo endoscopic sub score of 0–1; complete endoscopic remission was defined as a Mayo endoscopic sub score of 0. Follow up: 6 and 14 weeks after	At week 6, 55.8% (*n* = 43/77) achieved a clinical response and 18.2% (*n* = 14/77) achieved clinical remission. At week 14, 73.2% (*n* = 52/71) achieved a clinical response and 39.4% (*n* = 28/71) achieved clinical remission.

							vedolizumab initiation	
80	IBD	Brownson et al., 2022	United Kingdom	Retrospective cohort study	216 adult patients with inflammatory bowel disease	To describe the micronutrient status of a cohort of patients on biologic therapy for IBD and explore the relationship with disease activity	Faecal remission: C-reactive protein <10mg/L, albumin >35g/L and biochemical remission: faecal calprotectin (FCP) <250µg/g) at 12 months following biologic therapy	128 patients had Crohn's disease (CD). 97 patients were in biochemical remission at enrolment
50	IBD	Cohen et al., 2022	United States	Retrospective cohort study	119 patients (mean age: 39.4 years) with inflammatory bowel disease (IBD) who initiated tofacitinib therapy	To assess long-term effectiveness and safety of tofacitinib, effect on endoscopic remission rates, histologic changes, and alterations in faecal calprotectin levels	Clinical remission: Partial Mayo Score (PMS)<2; a score of 0 or 1 on the Mayo Endoscopic Score (MES)was regarded as endoscopic remission at weeks 8, 24, and 52	Clinical response and remission were observed in 70% and 21%, 59% and 33%, and 49%, and 37% at weeks 8, 24, and 52, respectively. Endo-histologic healing was achieved by 11%, 25%, and 37.5% of patients at weeks 8, 24, and 52, respectively.

51	IBD	Chugh et al., 2022	United States	Retrospective review	15 patients with Corhn's disease on upadacitinib	To assess the efficacy and safety of upadacitinib in this group of medically refractory patients with CD	Clinical remission was defined as average daily stool frequency of 1.5 and abdominal pain score of 1.0 at most recent follow-up, with neither worse than the baseline value	Of the 13 patients with follow up, 6 (46%) achieved clinical remission and 9 (69%) had a clinical response.
52	IBD	Dalal et al., 2022	United States	Retrospective cohort study	73 patients initiated tofa	To assess clinical outcomes up to 78 weeks after tofa initiation for UC in a real-world setting	steroid-free clinical remission (SFCR; i.e. simple clinical colitis activity index <=2 or per provider global assessment and no use of oral/IV corticosteroids for >=30 days) at 12, 52, and 78 (+/-4) weeks. Endoscopic remission: remission (Mayo endoscopic sub score 0) at >8 weeks, biochemical remission (normalized CRP or FC) at >8 weeks	31/60 (51.7%) were in steroid free clinical remission at 78 weeks and 21/ 47 (44.7%) achieved endoscopic remission (median time to endoscopy 58.1 weeks, IQR 30.7-103.6 weeks after initiation)

53	IBD	Dalal et al., 2022	United States	Retrospective cohort study	123 Crohn's disease and 34 Ulcerative colitis patients	To assess clinical and endoscopic outcomes at 12 and 24 months after ustekinumab (UST) dose intensification to every 4 weeks (q4w) or every 6 weeks (q6w) among patients with CD and UC	Corticosteroid-free clinical remission (CFCR; i.e. Harvey-Bradshaw index [HBI] <5 or simple clinical colitis activity index [SCCAI] <=2 or provider global assessment and no use of oral corticosteroids for >=30 days) at 12 (+/- 1) months post intensification.	For Crohn's disease, small bowel involvement (OR 2.42, p=0.049) was positively associated with CFCR at 12 months and opioid use (OR 0.36, p=0.014) and HBI (OR 0.77, p<0.001) were negatively associated with CFCR at 12 months. For ulcerative colitis, extraintestinal manifestation (OR 0.23, p=0.044) was negatively associated with CFCR at 12 months
115	IBD	Han et al., 2022	Unclear	Retrospective cohort study	50 Crohn's disease patients who had vedolizumab therapy	To investigate factors which have impact on earlier clinical remission and loss of response	Clinical remission defined as Crohn's Disease Activity Index under 150 without concomitant steroid use.	30 patients had dose intensification and 15 patients reached their first steroid- free clinical remission after dose intensification
91	IBD	McGrory et al., 2022	Australia	Retrospective review	57 patients who had received methotrexate	To audit the use of methotrexate with regard to prevalence of use, tolerability, clinical efficacy, and use of surveillance for liver fibrosis	Clinical remission of Crohn's disease defined as Harvey Bradshaw Index < 4; Clinical remission of ulcerative colitis defined as partial Mayo score<3	32/38 (84%) Crohn's disease patients and 8/9 (89%) ulcerative colitis patients were in clinical remission at last follow up;

81	IBD	Meade et al., 2022	United Kingdom	Retrospective cohort study	50 patients with Crohn's disease	To assess whether the Selecting Therapeutic Targets in Inflammatory Bowel Disease (STRIDE-II) endoscopic endpoints are achievable and whether the degree of mucosal healing (MH) affects long term outcomes	Combined remission (Simple Endoscopic Score for CD (SES-CD) =<2 and Harvey Bradshaw Index [HBI] <5) at follow up (median 39.9 months)	Combined remission occurred in 25 cases (50%)
54	IBD	Zeina et al., 2024	United States	Cohort study	139 patients with ulcerative colitis in deep remission	To evaluate for potential predictors and aetiologies of clinical relapse among patients with ulcerative colitis in deep remission.	Endoscopic remission (Mayo Endoscopic Score of 0 or 1) and histologic remission (Simplified Geboes Score <= 0.2) at follow-up (median 40 months)	Patients in deep remission have a 1-year risk of clinical relapse of <10%, with those demonstrating a non-normalized mucosa or elevated C-reactive protein predictive of persistent relapse risk. Discontinuation of therapy or minor histologic changes may drive relapse among those in deep remission

99	IBD	Kazama et al., 2023	Japan	Retrospective cohort study	117 patients (median age: 45 years) with inflammatory bowel disease (IBD) who received IFX-biosimilar (IFX-BS)	To assess long-term efficacy and safety of IFX-BS in patients with Crohn's disease (CD) and ulcerative colitis (UC)	In patients with CD, clinical remission (CR) was defined as Crohn's disease activity index (CDAI) < 150 points. In patients with UC, CR was defined as a pMayo score of two points or fewer at 8 weeks, 54 weeks, 2 years, 3 years, and 5 years	For CD: CR rates at 30 weeks, 54 weeks, 2 years, 3 years, 4 years, and 5 years were 0.90 (95%CI: 0.76–0.96), 0.85 (0.62–0.95), 0.78 (0.52–0.92), 0.74 (0.44–0.91), 0.78 (0.50–0.93), and 0.77 (0.49–0.92), respectively. For UC: CR rates at 8 weeks, 54 weeks, 2 years, 3 years, and 5 years were 100% (6/6), 75.0% (3/4), 33.3% (1/3), 66.7% (2/3), and 50% (1/2), respectively. Remission maintenance and treatment persistence rates beyond two years.
107	IBD	Pokryszka et al., 2023	Austria	Retrospective cohort study	58 patients with Crohn's disease	To explore associations between serum Vedolizumab (VDZ) serum concentrations and clinical/biomarker-defined endpoints	Clinical remission: stool frequency ≤ 3 and abdominal pain ≤ 1; biomarker remission: Faecal calprotectin (fCP) values ≤ 150 µg/g at 12,26,and 52 weeks	Clinical remission was achieved in 51.7% (30/58), 49.1% (26/53) and 45.2% (24/53) cases at weeks 12, 26 and 52, respectively, with biomarker remission rates of 24.1% (14/58), 35.8% (19/53) and 27.6% (16/53).

111	IBD	Shehab et al., 2024	Kuwait	Retrospective review	422 patients with IBD at two tertiary care centres in Kuwait, Haya Alhabib Gastroenterology Center and Farwaniya Hospital	To evaluate the effectiveness of biologic therapies in achieving clinical and endoscopic outcomes in biologic-naïve patients with IBD.	Patients who did not receive any steroid courses after 6 weeks from starting the biologic were considered to be in corticosteroid-free remission. Endoscopic remission was defined as an endoscopic Mayo score of 0–1 for patients with ulcerative colitis and a simple endoscopic score for Crohn's disease (SES-CD) of 0–2 for Crohn's disease at 12 months follow-up	In patients with CD, endoscopic remission was attained in 51 (52%) of the patients on adalimumab, 38 (53%) of the patients on infliximab, 34 (56%) of the patients on ustekinumab, and 16 (51%) of the patients on vedolizumab. In patients with UC, endoscopic remission was attained in 40 (56%) of the patients on infliximab, 26 (61%) of the patients on adalimumab, 8 (55%) of the patients on ustekinumab, and 11 (53%) of the patients on vedolizumab.
92	IBD	Alshiwanna et al., 2024	Australia	Cross-sectional study	1725 people (median age: 37 years) with IBD	To evaluate the role of dose escalation Infliximab (DE IFX) and patient outcomes in a real-world cohort.	Faecal calprotectin (FCP) remission rate defined as FCP <250µg/g 12 months post DE	Faecal calprotectin (FCP) remission rate (FCP <250µg/g) was higher 12 months post DE

55	IBD	Anyanwu et al., 2024	United States	Retrospective cohort study	260 biologic-naïve adults (mean age: 42.6 years) with mild to severe ulcerative colitis	To evaluate the effectiveness and onset of action of vedolizumab as a first-line biologic in biologic-naïve patients with UC	Clinical remission: partial Mayo score < 2; corticosteroid-free remission (clinical remission and no longer receiving corticosteroids) at 3, 6, 9, and 12 months after vedolizumab treatment initiation	At 12 months, 149/188 patients (79.3%) who remained on vedolizumab had clinical remission. Of 109 patients who were receiving a corticosteroid at baseline and remained on vedolizumab at 12 months, 52.3% were corticosteroid-free and had remission.
56	IBD	Chugh et al., 2024	United States	Retrospective cohort study	89 patients (median age: 43.9 years) with exposure to risankizumab (RZA) versus ustekinumab (UST)	To examine the efficacy of RZA in UST exposed patients in the real-world	Endoscopic remission was defined as simple endoscopic score for Crohn's disease (SES-CD) of 0 at 12-20 weeks post induction	On UST, 43% achieved clinical response, 10% achieved endoscopic or sonographic remission, and 29% achieved endoscopic or sonographic response. In comparison, these same patients later went on to receive RZA with 76% clinical response, 38% achieving endoscopic or sonographic remission,
57	IBD	Dalal et al., 2024	United States	Retrospective cohort study	155 adults (median age 40) initiated on upadacitinib (upa) vs tofacitinib (tofa) for ulcerative colitis	To compare real-world outcomes of upadacitinib (upa) vs tofacitinib (tofa) for ulcerative colitis	Steroid free clinical remission defined as : Simple clinical colitis activity index (SCCAI) <2 points or partial Mayo score <2 points or provider global assessment of clinical remission and no use of oral corticosteroids at	Upa was associated with significantly higher odds of SFCR at 12 weeks (OR 2.3) and 52 weeks (OR 3.0) and non-significantly higher odds of endoscopic response (OR 1.2) and endoscopic remission (OR 2.2) vs tofa

							assessment (12 and 52 weeks)	
82	IBD	Gros et al, 2024	United Kingdo m	Retrospective cohort study	290 adult inflammatory bowel disease (IBD) patients	To assess factors affecting vedolizumab (VDZ) persistence including clinical, biochemical and faecal biomarker remission at 1, 3 and 5 years	Clinical remission, biochemical remission and faecal biomarker remission were defined as a partial Mayo ⩽ 1, CRP ⩽ 5 mg/L and FC < 250 µg/g, respectively at years 1, 3 and 5	Clinical remission (partial Mayo < 2) was 75.7% (171/226), 72.4% (157/217) and 70.2% (127/181) at years 1, 3 and 5, respectively.
58	IBD	Lieto et al., 2024	United States	Retrospective cohort study	83 patients with ulcerative colitis (age 18+)	To describe the real-world effectiveness and safety of ozanimod (OZA)	Clinical remission: partial Mayo score (PMS) <2 at 12 weeks and 6 months post treatment. Endoscopic remission: Mayo endoscopic score <=1 or absence of erosions/ulcerations	At 6 months, 51% (19/37) were in clinical remission. Endoscopic assessment was performed a mean 257 (SD 135) days after starting OZA. Endoscopic remission was observed in 55% of patients (17/31)

104	IBD	Molina Arriero et al., 2024	Spain	Retrospective cohort study	35 patients (mean age: 53 years)	To evaluate the long-term effectiveness of dose intensification of ustekinumab	Clinical remission was defined as HBI < 5; biochemical remission as FCP < 150 µg/g at 6 and 12 months	At 6 months after UST intensification, 82.86% (n=29) of the patients presented clinical remission, with a statistically significant decrease in FCP levels (247.78 µg/g vs. 133.55 µg/g (p = 0.0026)). At 12 months, 85.71% had achieved clinical remission
97	IBD	Mulinacci et al., 2024	Italy	Retrospective cohort study	12 patients with Crohn's disease	To evaluate the safety and effectiveness of retreatment with subcutaneous infliximab vs Intravenous (IV) infliximab (IFX)	Steroid free remission: HBI≤4 at 6 months	At 6 months steroid free remission rate (HBI≤4) was 66%.
98	IBD	Parigi et al., 2024	Italy	Retrospective cohort study	679 patients with moderate-to-severe IBD	To assess the prevalence, characteristics, management, and outcomes of Difficult-to-treat (D2T) IBD	Biochemical remission (calprotectin <150 µg/g), and 13% in endoscopic remission (Mayo≤1; or SES-CD≤3. Clinical remission not defined	61% of patients with D2T-IBD were in clinical remission, 43% in biochemical remission (calprotectin <150 µg/g), and 13% in endoscopic remission (Mayo≤1; or SES-CD≤3 or Rutgeerts i0-i1)
59	IBD	Scalzo et al., 2024	United States	Retrospective cohort study	146 patients aged 18+ years	To describe the real-world effectiveness of Ozanimod (OZA) based on prior advanced therapy (AT) exposure	Clinical remission (CR) at 12 weeks, defined as partial Mayo score (PMS) < 2	36.1% (13/36) of patients with 3 or more AT exposures were in clinical remission at week 12

89	IBD	Xie et al., 2024	China	Retrospective cohort study	117 patients (median age: 32 years) with inflammatory bowel disease (IBD)	To evaluate the prognostic value of body composition-related imaging parameters in assessing Crohn's disease (CD) severity and biological responses	Clinical remission: CD Activity Index (CDAI) score<150; endoscopic remission, Simple Endoscopic Score for CD (SES-CD) ≤2 and no ulceration at week 48	The rates of both clinical remission and response were comparable between the sarcopenia and non-sarcopenia groups (remission: 68.2% vs. 72.7%, P=.77,
93	IBD	Chu et al. 2025	Australia	Retrospective cohort study	108 adults (18+ years) with a formal diagnosis of Crohn's disease	To evaluate Exclusive Enteral Nutrition (EEN) therapy in adults with Crohn's disease (CD) to identify determinants of clinical efficacy, adherence, and therapy completion	Clinical remission was defined by an HBI score ≤ 4	Among 80 patients treated for remission induction, remission and response rates were 50.0% (40/80) and 48.8% (39/80),
60	IBD	Gold et al., 2025	United States	Retrospective cohort study	201 patients with IBD prescribed a glucagon like peptide-1 agonists (GLP-1a)	To describe the tolerability and efficacy of GLP-1a therapy in patients with IBD	Clinical remission (defined as Harvey Bradshaw Index < 5 or partial Mayo < 2) at 3, 6, and 12 months after GLP-1a initiation	Clinical remission rates increased significantly from baseline (93% vs. 74%, P<0.01). Rates of clinical remission remained improved at the 6-month (81%, P=0.3) and 12-month (89%, P=0.09) time points.

90	IBD	Tu et al., 2025	China	Retrospective cohort study	183 patients with UC (median age: 40.9)	To identify independent factors associated with long-term outcomes,	Clinical remission: Simple Clinical Colitis Activity Index (SCCAI) score of ≤ 2; Endoscopic remission: Mayo Endoscopic Sub score (MES)score of ≤ 1 at follow-up (median follow-up: 5 years)	Histological remission showed fair agreement with endoscopic remission and independently predicted better clinical outcomes
83	IBD	Colwill et al., 2025	United Kingdom	retrospective review	51 patients (19+ years)	To assess if in those with uncontrolled disease on ustekinumab, switching to risankizumab is effective	Clinical remission: Harvey-Bradshaw Index (HBI)<5 at 3, 6, and 9 months	HBI decreased significantly at all timepoints (*P* < 0.001), with clinical remission rates increasing from 37.1% at baseline to 94.4% at 9 months
61	Type 2 Diabetes	Arterburn et al., 2013	United States	Retrospective cohort study	63717 severely obese adults (18-79 years old) with uncontrolled or medication-controlled diabetes who underwent bariatric surgery or received usual medical care from 2005 to 2008 in three health care delivery systems in the United States	To compare rates of diabetes remission, relapse and all-cause mortality among severely obese individuals with diabetes who underwent bariatric surgery vs. nonsurgically treated individuals	Remission defined as co-occurrence of (a) diabetes medication discontinuation (absence of pharmacotherapy for diabetes for ≥90 days after last prescription end date) and (b) control of T2DM (fasting glucose <126 and/or HbA1c <7% occurring ≥90 days after last prescription end date).	Age, site, duration of diabetes, haemoglobin A1c level, and intensity of diabetes medication treatment were significantly associated with remission. Bariatric subjects also experienced lower relapse rates than nonsurgical subjects (adjusted HR: 0.19; 95% CI: 0.15–0.23) with no higher risk of death (adjusted HR: 0.54; 95% CI: 0.22–1.30).

62	Type 2 Diabetes	Still et al., 2014	United States	Retrospective cohort study	690 patients (age range: <40 - 60+) who underwent RYGB surgery at the Geisinger Health System in Danville (PA, USA)	To develop a way to predict probability of diabetes remission after Roux-en-Y gastric bypass (RYGB) surgery on the basis of preoperative clinical criteria.	Partial remission was defined as HbA1c concentration of less than 6·5% (48 mmol/mol), fasting blood glucose concentrations of less than 7·0 mmol/L, and no use of antidiabetic drugs for at least 12 months. Complete remission was defined as HbA1c concentration of less than 6·0% (42 mmol/mol), fasting blood glucose concentrations of less than 5·55 mmol/L, and no use of antidiabetic drugs for at least 12 months	463 (63%) had achieved partial or complete remission

63	Type 2 Diabetes	Wood et al., 2015	United States	Retrospective cohort study	690 Type 2 diabetes patients (mean age: 47.9 years)	To determine the effects of incretins on type 2 diabetes (T2D) remission after Roux-en-Y gastric bypass (RYGB) surgery for patients taking insulin	Patients in "partial" remission were free of any use of antidiabetic medications, their fasted blood glucose levels were less than 125 mg/dL, and their HbA1c was less than 6.5% for a minimum of 12 months after surgery. Patients in "complete" remission had normal measures of glucose metabolism (ie, HbA1c < 5.7%, fasting glucose < 100 mg/dL) for at least 12 months after surgery, in the absence of active pharmacological therapy	Use of insulin, along with other antidiabetic medications, significantly diminished overall T2D remission rates 14 months after RYGB surgery (9%) compared with patients not taking insulin (56%). Addition of the GLP-1 agonist, however, increased significantly T2D early remission rates (22%), compared with patients not taking the Glucagon-like peptide 1 (GLP-1)GLP-1 agonist (4%).
84	Type 2 Diabetes	Gulliford et al., 2016	United Kingdom	Retrospective cohort study	826 obese participants (mean age: 50 years) with Type 2 diabetes who received bariatric surgery	To evaluate the effect of gastric banding, gastric bypass and sleeve gastrectomy on medium to long-term diabetes control in obese participants with type 2 diabetes mellitus	Remission was considered if the maximum HbA1c value recorded in one year was <6.5 % and there were no diabetes prescriptions issued in the same year	The proportion of patients in remission was 30 % in the second year. Rates of remission were maintained into the sixth year of follow-up

101	Type 2 Diabetes	Sheikh et al., (2017)	New Zealand	Cohort study	156 patients (18+) undergoing bariatric surgery between August 2005 and January 2008	To assess long-term results from a cohort of patients undergoing a silastic ring laparoscopic mini-gastric bypass SR-MGBP in a single centre	Resolution was determined by medication use from patient medication profiles from computer pharmacy dispensing records at 11 years	Comorbidity resolution, determined by medication use, showed a reduction in diabetes (21.8% to 7.1%), hypertension (37.2% to 21.4%) and hypercholesterolaemia (40.4% to 13.4%).
64	Type 2 Diabetes	Zaman et al., 2017	United States	Retrospective cohort study	155 morbidly obese patients undergoing bariatric surgery	To examine management of perioperative glucose levels in diabetic patients undergoing bariatric surgery and determined the impact of optimal glucose control	Diabetes resolution was defined as HgBA1C < 6.5 and absence of antihyperglycemic medications at 1 year follow-up	Diabetes resolution at 1 year was 70.1 % (73.4 % for RYGB and 53.9 % for LSG, p = 0.191). . In total, 96.7 % of optimally controlled patients experienced diabetes resolution at 1 year compared to 53.2 % in the non-optimally controlled group (p < 0.001).
65	Type 2 Diabetes	Chang et al., 2018	United States	Retrospective cohort study	3676 adults with type 2 diabetes	To assess if metabolic surgery counselling results in a significant increase in DM remission and A1c improvement	HbA1c<6.5% and no diabetes medications besides metformin at 1 and 3 year follow-up	In multivariable analyses adjusted for patient demographics and comorbidities, MS counselling was associated with an OR of 1.43 (95% CI 1.11-1.84; p=.0051) for achieving DM remission and an OR of 1.44 (95% CI 1.15-1.81; p=.0015) for achieving A1c control

66	Type 2 Diabetes	Still et al., 2019	United States	Retrospective cohort study	307 patients (mean age: 51.2 years) with Type 2 diabetes who had Roux-en-Y gastric bypass (RYGB) surgery	To determine whether adding duration of diabetes as an additional component of the DiaRem improves its ability to discriminate between patients with or without diabetes remission and/or reclassify pre-surgery patients into appropriate risk groups	Remission defined as haemoglobin A1c< 6.5% without diabetes medication occurring for one year, starting within two months of surgery	Diabetes remission occurred in 44% of patients and was strongly associated with both the DiaRem (po0.0001) and duration of diabetes (p o0.0001). Self-reported duration of diabetes was an acceptable surrogate for diabetes duration derived from clinical data. Diabetes duration of 4-10 years was associated with decreased chance of remission and duration of 5 years was associated with greater chance of remission. When duration of diabetes is available, DiaRem2 could be utilized as an alternative to DiaRem for evaluating likelihood of diabetes remission

100	Type 2 Diabetes	Seki et al., 2021	Japan	Retrospective cohort study	78 patients (mean age: 47.9) with mildly obese type 2 diabetes in Japan	To compare glycaemic control 1 year after treatment in patients with mildly obese (body mass index 27.5–34.9 kg/m2) type 2 diabetes mellitus who underwent bariatric surgery (BS) to those who received medical treatment (MT) in Japan.	HbA1c <6.5% without diabetes medication at 1 year follow-up	The diabetes remission rate (glycated haemoglobin <6.5% without diabetes medication) at 1 year was 59.0% in the bariatric surgery group and 0.4% in the medical treatment group ($P < 0.0001$).
67	Type 2 Diabetes	Ghusn et al., 2023	United States	Cohort study	Patients (mean age: 51 years) with BMI≥ 50 kg/m2, Type 2 diabetes, and have undergone RYGB or SG at three tertiary referral centres in the United States	To study real-world T2DM long-term remission in patients with BMI ≥ 50 kg/m^2 following Roux-En-Y gastric bypass (RYGB) or sleeve gastrectomy (SG).	HbA1c < 6.5% without the use of any anti-diabetic medication for ≥ 5 years)	Long-term T2DM remission (≥ 5 years) was demonstrated in 47% of patients. The duration of T2DM ($p < 0.0001$), number of T2DM medications ($p = 0.003$) and weight loss ($p = 0.048$) were the only independent factors for long-term T2DM remission.

13	Type 2 Diabetes	Dambha-Miller et al., 2023	United Kingdo m	Retrospective cohort study	60,287 people with type 2 diabetes	To identify distinct diabetes remission trajectories in a large population-based cohort over seven-years follow-up and to examine associations between remission trajectories and diabetes complications	Remission was defined as having two HbA1c level < 48 mmol/mol (6.5%) measurements separated over a period of at least six months in the absence of diabetes medications or bariatric surgery	11,491 (19.1%) people achieved remission. Risk of CVD outcomes vary by pattern of remission over time, with lowest risk for those in remission longer.
102	Type 2 Diabetes	Witcomb Cahill et al, 2024	New Zealand	Retrospective cohort study	579 patients who received bariatric and metabolic surgery (BMS)	To examine the rates and outcomes of BMS between patients domiciled in a metropolitan versus provincial area in Aotearoa New Zealand	Diabetes remission was defined as an HbA1c dropping below 43mmol/mol within 6 months	There was a higher resolution of diabetes in the provincial patients.
112	Type 2 Diabetes	Ahmadi et al., 2025	Pakistan	Retrospective cohort study	81 patients (mean age 47.2 years)	To investigate the role of metabolic bariatric surgery (MBS) in achieving Type 2 diabetes	Complete remission defined as fasting blood glucose <100 mg/dL and HbA1c <5.7%, sustained for at least 1 year without the use of any glucose-lowering medications, and partial remission was defined as fasting glucose between 100 and 125 mg/dL and HbA1c <6.5%, sustained for at least 1 year without	By 12 months, complete remission was observed in 29 of 81 patients (35.8%).

							antidiabetic medications	
85	Type 2 Diabetes	Griffiths et al., 2025	United Kingdo m	observational pre-post quasi-experimental analysis	1,051 patients who attended at least one intervention session	To assess effectiveness of an intensive dietary support intervention (REWIND)	Remission defined as HbA1c <48 mmol/mol with no record of glucose lowering treatment for at least 90 days prior to the measurement	Participation was associated with a +4·2% (3·9, 4·5) increase in remission,
68	Type 2 Diabetes	Thapa et al., 2025	United States	Retrospective cohort study	556,758 adults (≥18 years) with type 2 diabetes	To assess the real-world frequency and characteristics associated with type 2 diabetes remission	Remission was defined as HbA1c <6.5% persisting for >=3 months after cessation of glucose-lowering medications	2.9% (16,016 adults) achieved type 2 diabetes remission, although 36.9% of those who experienced remission relapsed. The strongest characteristics associated with remission were not receiving glucose-lowering medications at baseline versus three or more medications (odds ratio [OR] 15.9, 95% CI 12.1-21.0), baseline HbA1c <7% vs. >=11% (OR 3.1, 2.9-3.3) and diabetes

								duration <1 year versus >=4 years (OR 2.6, 2.5-2.7)
69	Drug and/or alcohol misuse	Williams et al., 2017	United States	Retrospective review	2101 patients (18+) living with HIV (PLWH) with unhealthy alcohol use from	To assess whether PLWH who screened positive for unhealthy alcohol use were more likely to resolve unhealthy drinking one year later if they had brief alcohol intervention (BI) documented in their electronic health record	Resolution of unhealthy alcohol use was defined as screening negative on the next annual follow-up screening (AUDIT-C<5) with at least a 2-point reduction from the previous score at 1 year follow-up	61% resolved unhealthy alcohol use at follow-up. Documented brief intervention was not associated with resolution [Adjusted incidence rate ratio 0.96, (95% CI 0.90–1.02)].

110	Drug and/or alcohol misuse	Rautiainen et al., 2019	Finland	Prospective cohort study	396 working-aged (18–65 years) individuals had an alcohol-related visit to health services between the years 2011–2012	To examine the extent and continuity of alcohol-related social and health service utilization (HSU) of individuals with alcohol use disorder (AUD) and predicts longitudinal care outcomes	Remission was defined as sustained abstinence or managed use that lasted until the end of the follow-up period with minimum duration of six months	Institutional care (odds ratio [OR] 1.55), contact with mental health nurse (OR 1.22), and visits to the primary health care (PHC) doctor for MH reasons (OR 1.89) were associated with increased odds of remission.
70	Drug and/or alcohol misuse	Palzes et al., 2020	United States	Observational cohort study	4,078 adults (age 18- 65+) with alcohol use disorder (AUD) who screened positive for unhealthy drinking	To examine correlates of remission from unhealthy drinking among patients with an alcohol use disorder (AUD)	Remission defined as negative screening (National Institute on Alcohol Abuse and Alcoholism (NIAAA)) during follow-up (up to 3 years) when the patient reported abstinence or low-risk drinking (i.e., drinking within recommended daily and weekly limits),	The median time to remission from unhealthy drinking was 1.7 years. Factors significantly associated with greater odds of remitting from unhealthy drinking during follow-up were female gender; older age (50–64 years); Black or Latino/Hispanic race/ethnicity; having more medical comorbidities; not having a comorbid drug use disorder; lower alcohol consumption levels; and receiving addiction medicine treatment before the index screening

71	Drug and/or alcohol misuse	Zhou et al., 2021	United States	Retrospective case control	72.9 million patients	To present an integrated drug repurposing strategy that combines computational prediction, clinical corroboration	Remission defined based on “remission of OUD” was based on the diagnosis of “Opioid dependence in remission (disorder)” (SNOMED-CT concept code 191821007) (follow up :up to 21 years)	Tramadol, olanzapine, mirtazapine, bupropion, and atomoxetine were associated with increased odds of OUD remission (adjusted odds ratio: 1.51 [1.38–1.66], 1.90 [1.66–2.18], 1.38 [1.31–1.46], 1.37 [1.29–1.46], 1.48 [1.25–1.76], *p* value < 0.001, respectively).
105	Drug and/or alcohol misuse	Danisman et al., 2024	Turkey	Retrospective cross sectional study	92 individuals (mean age 29 years)	To investigate the remission rates of patients with opioid use disorder who got naltrexone implantation	Remission assumed if no opioid positivity detected in monthly urine analyses during active treatment and three months post treatment	Based on multiple Poisson regression analyses, the duration of the "longest period of not using heroin" was the predictor of "post-implantation remission duration".
72	Drug and/or alcohol misuse	Osterhage et al., 2025	United States	Cross-sectional study	307 patients	To describe application of ICD-10 diagnosis codes for opioid use, dependence and abuse from an electronic health record (EHR) data	Remission identified using ICD-10 remission codes	F11.21 [opioid dependence, in remission] was used more consistently for patients with OUD

73	Drug and/or alcohol misuse	Gao et al., 2025	United States	Retrospective cohort study	Amphetamine-type stimulant use disorders (ATSUD) patients who received anaesthesia (n=3663) or were diagnosed with depression (n=4328)	To assess the association between ketamine and ATSUD remission	Remission from ATSUD, the outcome of interest, was identified through the diagnosis of 'other stimulant abuse in remission' (ICD F15.11), 'other stimulant dependence in remission' (ICD F15.21) or 'other stimulant use unspecified in remission' (ICD F15.91. Follow- up: up to 3 years	There appears to be an association between clinician-prescribed ketamine and higher remission rates in patients with amphetamine-type stimulant use disorders
74	Drug and/or alcohol misuse	Hailer et al., 2025	United States	Retrospective cohort study	6945 females, aged 12- 49 years with a childbirth code	To evaluate the association of documented medication for opioid use disorder (MOUD) prescription during pregnancy with maternal outcomes	Remission identified using ICD-10 remission codes from 1 week to 1-3 years post childbirth	A documented prescription for MOUD during pregnancy is associated with newly documented remission of OUD

86	Multiple conditions	Ledwaba-Chapman et al., 2021	United Kingdom	Cross-sectional study	826,936 adults (≥18 years of age) registered between 2005 -2020 in general practices in one inner London borough	To estimate the prevalence and determinants of multimorbidity in an urban, multi-ethnic area over 15-years and investigate the effect of applying resolved/remission codes on prevalence estimates.	Classification of resolved/remission was based on clinical coding defined by the patient's general practitioner over 15 years	Asthma (53.2%) and depression (20.2%) were responsible for most resolved and remission codes. The study highlights the importance of applying resolved/remission codes to obtain an accurate prevalence
94	Multiple sclerosis	Zafar et al., 2021	Saudi Arabia	Retrospective cross-sectional study	119 patients (Aged 18- 50 years) with relapsing-remitting multiple sclerosis (RRMS)	To identify the prevalence of No evidence of disease activity (NEDA-3) status achievement in patients with RRMS on disease-modifying treatments (DMTs) (mainly the interferon) and to describe the factors affecting its attainment	No relapse, no disability progression, and no new (>3 mm)/enlarged T2-or Gdenhancing lesion at 1 year follow-up or more. Relapse defined as: The appearance of either a new neurological abnormality, or the worsening of a previously stable abnormality lasting for ≥24 h, in the absence of fever or infection within 1 week of symptom onset. Disability progression: An increase in the EDSS score of ≥1.5 points between two time points, if the baseline EDSS score was 0.0, ≥1.0 point if the	NEDA-3 status was achieved in 41 (33.6%) patients

							baseline EDSS score was 1.5, and ≥0.5 points if the baseline EDSS score was >5. Focal MRI activity: Development of new (>3 mm) or enlargement of established T2 lesions, and/ or Gd-enhanced T1 lesions	
95	Multiple sclerosis	Alshamrani et al., 2024	Saudi Arabia	Retrospective cohort study	231 patients (Aged 18- 50 years) with relapsing-remitting multiple sclerosis	To identify the response rate to oral disease-modifying treatments (oDMT) in patients with relapsing–remitting multiple sclerosis (PwRRMS) compared to interferon (IFN) in terms of achieving no evidence of disease activity-3 (NEDA-3	No relapse, no disability progression, and no new (>3 mm)/enlarged T2-or Gdenhancing lesion at 1 year follow-up or more. Relapse defined as: The appearance of either a new neurological abnormality, or the worsening of a previously stable abnormality lasting for ≥24 h, in the absence of fever or infection within 1 week of symptom onset. Disability progression: An increase in the EDSS score of ≥1.5 points between two time points, if the baseline EDSS score was 0.0, ≥1.0 point if the baseline EDSS score was 1.5, and ≥0.5 points if the baseline	More than half of the patients with relapsing–remitting multiple sclerosis on oral DMTs achieved NEDA-3 status

96	Multiple sclerosis	Alharbi et al., 2023	Saudi Arabia	Retrospective review	93 patients with multiple sclerosis	To explore the cost and consequence of ocrelizumab in managing relapsing remitting multiple sclerosis as a second-choice treatment	EDSS score was >5. Focal MRI activity: Development of new (>3 mm) or enlargement of established T2 lesions, and/ or Gd-enhanced T1 lesions No evidence of disease activity (NEDA-3) (i.e., absence of new T2 or T1 gadolinium (Gd) lesions as demonstrated by the Magnetic Resonance Imaging (MRI), disability progression(e.g.: increased walking difficulty leading to loss of independence), and clinical relapses(no return of old symptoms or worsening of current Multiple sclerosis symptoms (i.e., clinical relapse)) at 6 months+ follow-up	Rituximab seems to be more effective and is less costly than natalizumab in the management of relapsing remitting multiple sclerosis
