# Supplementary material for: Understanding Remission of Long-Term Conditions Through Electronic Health Records: Scoping Review
Source: J Med Internet Res. 2026 May 19;28:e80796. doi: 10.2196/80796 (PMC13186534; doi:10.2196/80796)
Supplement: Multimedia Appendix 2 [file jmir-v28-e80796-s002.pdf]

Search strategies for electronic databases

OVID Medline search strategy

1. exp electronic health records/
2. exp medical records systems, computerized/
3. (electronic or computeri#ed or digital or automated or “computer-stored” or “computer-based”) adj3 record*.mp
4. EHR.mp
5. EMR not electron magnetic resonance.mp
6. 1 or 2 or 3 or 4 or 5
7. exp Remission Induction/
8. (remission or resolution or (absence AND disease activ*) or remit* or "inactive disease").mp
9. 7 or 8
10. exp Anemia/ or anaemia.mp.
11. exp Anxiety Disorders/ or anxiety.mp.
12. asthma.mp. or exp Asthma/
13. chronic pancreatitis.mp. or exp Pancreatitis, Chronic/
14. Liver Disease.mp. or exp Liver Diseases/
15. exp Substance-Related Disorders/
16. chronic kidney disease.mp. or exp Renal Insufficiency, Chronic/
17. exp Depression/ or depression.mp.
18. diabetes.mp. or exp Diabetes Mellitus/
19. exp Endometriosis/ or endometriosis.mp
20. epilepsy.mp. or exp Epilepsy/
21. eating disorder.mp. or exp "Feeding and Eating Disorders"/
22. gout.mp. or exp Gout/
23. hypertension.mp. or exp Hypertension/
24. inflammatory bowel disease.mp. or exp Inflammatory Bowel Diseases/
25. exp Multiple Sclerosis/ or multiple sclerosis.mp.
26. peptic ulcer disease.mp. or exp Peptic Ulcer/
27. exp Peripheral Vascular Diseases/ or peripheral vascular disease.mp.
28. exp Stroke/ or stroke.mp.
29. exp Tuberculosis/ or tuberculosis.mp.
30. thyroid disease.mp. or Thyroid Diseases/
31. exp Ischemic Attack, Transient/ or Transient ischaemic attack.mp.
32. exp Urinary Tract Infections/ or chronic urinary tract infection.mp.
33. exp Vision Disorders/ or visual impairment.mp.
34. exp Chronic Disease/
35. ("chronic illness*" or "chronic disease*” or “chronic condition*” or “long-term disease” or “long-term illness” or “long-term condition”).mp

36. 10 or 11 or 12 or 13 or 14 or 15 or 16 or 17 or 18 or 19 or 20 or 21 or 22 or 23 or 24 or 25 or 26 or 27 or 28 or 29 or 30 or 31 or 32 or 33 or 34 or 35
37. 6 and 9 and 36
38. 37 not ((exp "Animals"/ or exp "Plants"/) not "Humans"/)

Embase search strategy

exp electronic health records/

(electronic or computeri#ed or digital or automated or "computer-stored" or "computer-based") adj3 record*.mp

EHR.mp

EMR not electron magnetic resonance.mp

1 or 2 or 3 or 4
exp remission/
(remission or "disease resolution" or (absence AND disease activit*) or remitted or "inactive disease").mp
exp anemia/ or anaemia.mp.
anxiety disorder.mp. or exp anxiety disorder/
asthma.mp. or exp asthma/
chronic pancreatitis.mp. or exp chronic pancreatitis/
exp liver disease/ or liver disease.mp.
substance related disorder.mp. or exp drug dependence/
alcohol dependence.mp. or exp alcoholism/
exp chronic kidney failure/ or chronic kidney disease.mp.
exp chronic depression/ or depression.mp.
diabetes.mp. or exp diabetes mellitus/
exp endometriosis/ or endometriosis.mp.
epilepsy.mp. or exp epilepsy/
exp eating disorder/ or eating disorder.mp.
exp gout/ or gout.mp.
exp hypertension/ or hypertension.mp.
exp Crohn disease/ or exp ulcerative colitis/ or inflammatory bowel disease.mp. or exp inflammatory bowel disease/
exp multiple sclerosis/ or multiple sclerosis.mp.
exp peptic ulcer/ or peptic ulcer.mp.
exp peripheral vascular disease/ or peripheral vascular disease.mp.
exp cerebrovascular accident/ or stroke.mp.
exp tuberculosis/ or tuberculosis.mp.

exp thyroid disease/ or thyroid disease.mp.

exp transient ischemic attack/ or transient ischaemic attack.mp.

exp urinary tract infection/ or urinary tract infections.mp.

exp visual impairment/ or visual impairment.mp.

("chronic illness*" or "chronic disease*" or “chronic condition*” or “long-term disease” or “long-term illness” or “long-term condition”).mp

exp chronic disease/

6 or 7

8 or 9 or 10 or 11 or 12 or 13 or 14 or 15 or 16 or 17 or 18 or 19 or 20 or 21 or 22 or 23 or 24 or 25 or 26 or 27 or 28 or 29 or 30 or 31 or 32 or 33 or 34

5 and 35 and 36

37 not ((exp "Animals"/ or exp "Plants"/) not "Humans"/)

Limit 38 to (“remove medline records”)

Cochrane Library

#1 MeSH descriptor: [Medical Records Systems, Computerized] explode all trees
#2 MeSH descriptor: [Database Management Systems] explode all trees

#3 #1 or #2

#4 (remission or resolution):ti,ab,kw (Word variations have been searched)

#5 (absence AND “disease activity”):ti,ab,kw (Word variations have been searched)

#6 (disease inactivity):ti,ab,kw (Word variations have been searched)

#7 #4 or #5 or #6

#8 MeSH descriptor: [Chronic Disease] explode all trees

#9 MeSH descriptor: [Generalized Anxiety Disorder] explode all trees

#10 MeSH descriptor: [Asthma] explode all trees

#11 MeSH descriptor: [Pancreatitis, Chronic] explode all trees

#12 MeSH descriptor: [Liver Diseases] explode all trees

#13 MeSH descriptor: [Substance-Related Disorders] explode all trees

#14 MeSH descriptor: [Anemia] explode all trees

#15 MeSH descriptor: [Renal Insufficiency, Chronic] explode all trees

#16 MeSH descriptor: [Depression] explode all trees

#17 MeSH descriptor: [Diabetes mellitus] explode all trees

#18 MeSH descriptor: [Endometriosis] explode all trees

#19 MeSH descriptor: [Epilepsy] explode all trees

#20 MeSH descriptor: [Feeding and Eating Disorders] explode all trees

#21 MeSH descriptor: [Gout] explode all trees

#22 MeSH descriptor: [Hypertension] explode all trees

#23 MeSH descriptor: [Inflammatory Bowel Diseases] explode all trees

#24 MeSH descriptor: [Multiple Sclerosis] explode all trees

#25 MeSH descriptor: [Peptic Ulcer] explode all trees

#26 MeSH descriptor: [Peripheral Vascular Diseases] explode all trees

#27 MeSH descriptor:[Stroke] explode all trees

#28 MeSH descriptor: [Tuberculosis] explode all trees

#29 MeSH descriptor: [Thyroid Diseases] explode all trees

#30 MeSH descriptor: [Urinary Tract Infections] explode all trees

#31 MeSH descriptor: [Vision Disorders] explode all trees

#32 (anemia or anaemia or anxiety disorder* or asthma or chronic pancreatitis or chronic liver disease or substance related disorder* or alcohol dependency or chronic kidney disease or depression or diabetes or endometriosis or epilepsy or eating disorder or gout or hypertension or inflammatory bowel disease or multiple sclerosis or peptic ulcer disease or peripheral vascular disease or stroke or tuberculosis or thyroid disease or transient ischaemic attack or urinary tract infection or visual impairment):ti,ab,kw (Word variations have been searched)

#33 #8 or #9 or #10 or #11 or #12 or #13 or #14 or #15 or #16 or #17 or #18 or #19 or #20 or #21 or #22 or #23 or #24 or #25 or #26 or #27 or #28 or #29 or #30 or #31 or #32

#3 and #7 and #33

CINAHL EBSCO

S1 MH (electronic health record* or electronic medical record* or emr or ehr)

S2 XB (datasets and database in healthcare)

S3 XB ((((computeri?ed or electronic* or digital or automated) N3 ("record#" or "record#"))))

S4 XB (electronic health record* or electronic medical record* or emr or ehr)

S5 XB medical records systems, computerized

S6 S1 or S2 or S3 or S4 or S5

S7 MH (remission or recovery or reversal)

S8 MH (chronic disease or chronic illness or long-term conditions or chronic conditions)

S9 MH (anemia or anaemia or anxiety disorder* or asthma or chronic pancreatitis or chronic liver disease or substance related disorder* or alcohol dependency or chronic kidney disease or depression or diabetes or endometriosis or epilepsy or eating disorder or gout or hypertension or inflammatory bowel disease or multiple sclerosis or peptic ulcer disease or peripheral vascular disease or stroke or tuberculosis or thyroid disease or transient ischaemic attack or urinary tract infection or visual impairment)
S10 S8 or S9

S11 S6 and S7 and S10

S12 Limiters - Exclude MEDLINE records
S13 (MH "Animals+") NOT (MH "Human")
S14 S11 NOT S13

Bielefeld Academic Search Engine

Basic Search

Subject: electronic medical records remission chronic disease (Additional word forms)

Subject: electronic medical records remission long-term conditions (Additional word forms)
